# Genomic assessment of local adaptation in dwarf birch to inform assisted gene flow

**DOI:** 10.1111/eva.12883

**Published:** 2019-11-24

**Authors:** James S. Borrell, Jasmin Zohren, Richard A. Nichols, Richard J. A. Buggs

**Affiliations:** ^1^ Jodrell Laboratory Royal Botanic Gardens, Kew Surrey UK; ^2^ Sex Chromosome Biology Lab The Francis Crick Institute London UK; ^3^ School of Biological and Chemical Sciences Queen Mary University of London London UK

**Keywords:** adaptive potential, assisted gene flow, climate change, conservation genetics, environmental association analysis, evolutionary conservation, landscape genomics, provenance matching

## Abstract

When populations of a rare species are small, isolated and declining under climate change, some populations may become locally maladapted. Detecting this maladaptation may allow effective rapid conservation interventions, even if based on incomplete knowledge. Population maladaptation may be estimated by finding genome–environment associations (GEA) between allele frequencies and environmental variables across a local species range, and identifying populations whose allele frequencies do not fit with these trends. We can then design assisted gene flow strategies for maladapted populations, to adjust their allele frequencies, entailing lower levels of intervention than with undirected conservation action. Here, we investigate this strategy in Scottish populations of the montane plant dwarf birch (*Betula nana*). In genome‐wide restriction site‐associated single nucleotide polymorphism (SNP) data, we found 267 significant associations between SNP loci and environmental variables. We ranked populations by maladaptation estimated using allele frequency deviation from the general trends at these loci; this gave a different prioritization for conservation action than the Shapely Index, which seeks to preserve rare neutral variation. Populations estimated to be maladapted in their allele frequencies at loci associated with annual mean temperature were found to have reduced catkin production. Using an environmental niche modelling (ENM) approach, we found annual mean temperature (35%), and mean diurnal range (15%), to be important predictors of the dwarf birch distribution. Intriguingly, there was a significant correlation between the number of loci associated with each environmental variable in the GEA and the importance of that variable in the ENM. Together, these results suggest that the same environmental variables determine both adaptive genetic variation and species range in Scottish dwarf birch. We suggest an assisted gene flow strategy that aims to maximize the local adaptation of dwarf birch populations under climate change by matching allele frequencies to current and future environments.

## 1 INTRODUCTION

Climate change is predicted to become a major driver of global biodiversity loss (Bellard, Bertelsmeier, Leadley, Thuiller, & Courchamp, 2012; Urban, 2015). Species that lack relevant phenotypic plasticity (Gratani, 2014; Nicotra et al., 2010) may survive environmental changes by dispersing to new locations, consequently tracking conditions they are currently adapted to (Aitken, Yeaman, Holliday, Wang, & Curtis‐McLane, 2008; Meier, Lischke, Schmatz, & Zimmermann, 2012), or remaining in the same location and rapidly evolving adaptation to their new environments from standing genetic variation or gene flow (Aitken et al., 2008; Alberto et al., 2013). Migration in response to rapid climate change may be particularly difficult for plants (Corlett & Westcott, 2013; Hampe & Petit, 2005; Zhu, Woodall, & Clark, 2012). In some cases, plants lack the dispersal ability to keep pace with accelerated climate shifts (Loarie et al., 2009). For example, there may be an absence of potential habitat at higher latitudes (McKenney, Pedlar, Lawrance, Campbell, & Hutchinson, 2007) or altitudes (Engler et al., 2011), suitable new habitats may be separated by too large distances (Meier et al., 2012) or dispersal may be impossible due to anthropogenic habitat fragmentation. In these cases, conservation managers aiming to prevent extinction of species or populations face a choice between relying on in situ evolution to track the environmental change or attempting conservation interventions such as assisted migration or assisted gene flow (AGF) that seeks to enable, facilitate or accelerate adaptation.

To evaluate whether interventions are appropriate, a first step is understanding current local adaptation and the potential for adaptation to future environments (Davis, Shaw, & Ettersonm, 2005; Funk, Forester, Converse, Darst, & Morey, 2019; Hoffmann, Sgrò, & Kristensen, 2017). The classical way to identify local adaptation is via reciprocal transplant experiments (Kawecki & Ebert, 2004; Leimu & Fischer, 2008; Pardo‐Diaz, Salazar, & Jiggins, 2015). However, this approach is often unfeasible for wild organisms with long generation times in need of urgent conservation, meaning that more rapid approaches using genomics are desirable (Williams et al., 2008).

Genotype–environment association (GEA; also referred to as environmental association analysis, EAA) methods are increasingly used to identify loci involved in local adaptation (Abebe, Naz, & Léon, 2015; Ahrens et al., 2018; Bay, Rose, Logan, & Palumbi, 2017; Coop, Witonsky, Rienzo, & Pritchard, 2010; Flanagan, Forester, Latch, Aitken, & Hoban, 2018; Funk et al., 2019; Godbout, Gros‐Louis, Lamothe, & Isabel, 2019; Günther & Coop, 2013; Ingvarsson & Bernhardsson, 2019; Mahony et al., 2019; Rellstab, Gugerli, Eckert, Hancock, & Holderegger, 2015). These approaches detect replicated signatures of selection (single nucleotide polymorphisms [SNPs] that deviate strongly from estimated neutral population structure) across many independent populations. Thus far, the majority of studies to apply GEA in tree species have been targeted at candidate genes and surveyed fewer than 350 loci (Keller, Levsen, Olson, & Tiffin, 2012; Nadeau, Meirmans, Aitken, Ritland, & Isabel, 2016; Rellstab et al., 2016; Wang, Wang, Xia, & Su, 2016) though three other studies using larger numbers of loci are presented in this journal issue (Godbout et al., 2019; Ingvarsson & Bernhardsson, 2019; Mahony et al., 2019).

Building on the assumption that GEA captures an important component of locally adaptive allelic variation, especially if based on genome‐wide markers, we may extend it to rapidly assess local adaptation and adaptive potential within populations. The principal behind this approach is the detection of discordance between genotype and environment, in certain populations, as an indicator of reduced local adaptation and vulnerability to future demographic decline (Alberto et al., 2013). In a previous study, Rellstab et al. (2016) developed a model to estimate the average change in allele frequency at environmentally associated loci that would be required to respond to projected future environmental conditions. They based this estimate on the allele frequency changes that would maintain the present‐day associations between genotype and environment and termed this mismatch, the risk of nonadaptedness (RONA). For clarity, we term this “future risk of nonadaptedness” (f‐RONA) and comment that rather than a “risk” this is a forecast, but for consistency, we maintain the same terminology in this manuscript. This approach to estimating adaptation has many simplifying assumptions. Environmental variation in nature is complex, as are the mechanisms by which organisms adapt to them, but as Funk et al. (2019) argue, any available evidence may improve conservation decision‐making.

Here, we extend the work of Rellstab et al. (2016) to explicitly define c‐RONA, the “current risk of nonadaptedness,” that is the average change in allele frequency at climate‐associated loci required to match our estimate of the optimum for current climatic conditions (for a given environmental factor). Current risks are likely to be particularly important for species that are already declining due to climate change and have small isolated populations. Furthermore, we extend the univariate RONA model to a multi‐locus analysis of genome‐wide markers and use best linear unbiased prediction (BLUP) to improve our estimate of the effect of each locus.

In populations where c‐RONA is high, local genotypes would not match local environmental variables as expected. Therefore, a possible management intervention is to use AGF to introduce more appropriate alleles or adjust population allele frequencies. Here, AGF is defined as the managed movement of individuals or gametes between populations, from source populations that have been selected with the aim of accelerating adaptation, so that it is faster than would occur by passive natural dispersal alone (Aitken & Whitlock, 2013). This AGF strategy could be used to inform sourcing of seed stock for reforestation programmes (Boshier et al., 2015) and mitigate maladaptation to future climate (Aitken & Bemmels, 2016; Havens et al., 2015; Jin et al., 2016). Importantly, only modest translocation of genotypes may enhance adaptation by introducing genetic variation upon which selection can act to further refine local allele frequencies (Bay et al., 2017; Pavlova et al., 2017). Conversely, such interventions could have negative effects (i.e., outbreeding depression) if they cause gene flow between populations with undetected adaptive differentiation (Frankham et al., 2011; Pavlova et al., 2017). We note that where target populations are small, maladapted and dominated by drift, AGF is equivalent to genetic rescue (see Aitken & Whitlock[, 2013] for a detailed review).

If AGF is to be effective, there must be appropriate populations from which to source migrants. Such populations might be found towards the species’ retreating range edge or other locations where environmental conditions are closer to those anticipated in the future (Olson et al., 2013). To design a sampling strategy that encompasses both environmental gradients and declining range edge populations threatened by environmental change, we can use environmental niche models (ENMs; Maguire, Nieto‐Lugilde, Fitzpatrick, Williams, & Blois, 2015). ENMs project the distribution of species’ ranges under current and future climate scenarios based on observation data and can guide effective sampling (Elith & Leathwick, 2009). ENMs are also an established tool for conservation practitioners seeking to understand major climatic selection pressures and projected range shifts for threatened species, but often lack integration and comparison with genomic assays of local adaptation (Hällfors et al., 2015; Razgour et al., 2019).

Here, we conduct GEA and ENM analysis of wild populations of dwarf birch (*Betula nana*), for which we have field observation and genome‐wide population genetic data. In the UK, dwarf birch is a nationally scarce montane tree that has experienced an accelerated decline in recent decades, likely due to the combined impact of anthropogenic climate change and moorland management that permits over‐browsing and burning (Aston, 1984; Borrell, Wang, Nichols, & Buggs, 2018; Wang et al., 2014; Zohren et al., 2016). Dwarf birch, like many tree species, is the focus of a conservation programme to restore populations, delimit management units and prioritize the protection of important genetic diversity (Koskela et al., 2013). Germplasm collection from central Scottish Highland populations is already underway for reintroduction to other parts of the species former range (J Borrell pers. obs.). Previous research by our group has found that despite extensive fragmentation, most populations of dwarf birch in the UK contain diversity comparable to that of large, unfragmented Scandinavian populations (Borrell et al., 2018). Nevertheless, we concluded that this diversity has become increasingly partitioned among populations. In other words, much of the adaptive diversity in dwarf birch is still extant in the UK, but due to restricted gene flow and dispersal, marginal populations may be maladapted due to a failure to track environmental change, or by drift of adaptive alleles away from their optimum frequency. There is limited potential for naturally occurring gene flow to enhance future adaptation in many populations.

In species subject to conservation management such as dwarf birch, evolutionary processes have sometimes been overlooked, despite the importance of adaptation to species persistence (Eizaguirre & Baltazar‐Soares, 2014; Fitzpatrick & Keller, 2015). Therefore, the adaptive potential of populations may be underrepresented in conservation prioritization strategies (Funk et al., 2019; Harrisson, Pavlova, Telonis‐Scott, & Sunnucks, 2014). For example, where genetic diversity information is available to conservationists, metrics that score populations on neutral genetic distinctiveness, such as the Shapley index, are often used (Haake, Kashiwada, & Su, 2007; Isaac, Turvey, Collen, Waterman, & Baillie, 2007; Volkmann, Martyn, Moulton, Spillner, & Mooers, 2014). However, there is no guarantee that neutral and adaptive diversity will be correlated (Bonin, Nicole, Pompanon, Miaud, & Taberlet, 2007), and indeed, approaches designed solely to promote or conserve neutral diversity may be harmful (Reed & Frankham, 2003; Weeks, Stoklosa, & Hoffmann, 2016). Therefore, evaluating adaptive diversity, rather than using more established metrics of genetic diversity, should improve the prioritization decisions in species management, though see Kardos and Shafer (2018) for potential pitfalls.

To explore potential management strategies for dwarf birch, that takes into account local adaptation and evolutionary potential, we first characterize the species’ range using ENMs under present and projected future climate scenarios. We evaluate these ENMs by assessing whether populations on the margins of the inferred distribution had lower scores for phenotypic and fitness proxies for local adaptation. Second, we use GEA to survey putative adaptive loci across the species’ range and estimate c‐RONA to identify populations with a discordance between genotype and environment. The combined ENM and GEA data present an opportunity to test the hypothesis that limiting environmental variables (which have higher discriminatory power in an ENM) have more genomic loci associated with them in GEA, perhaps as a result of stronger selection for adaptation (an alternative would be that certain variables limit species’ ranges precisely because they lack genetic adaptation). We provide preliminary evidence in support of this hypothesis in dwarf birch. Third, we evaluate our estimates of nonadaptedness (c‐RONA) of dwarf birch populations against the Shapley index, an existing conservation prioritization most often applied to neutral markers. Finally, we illustrate a strategy of AGF to maximize adaptive genetic diversity and hence sustain the adaptive potential of British dwarf birch populations. We discuss the advantages and limitations of this approach in the context of managing dwarf birch and other plants exposed to rapid environmental change.

## 2 METHODS

### 2.1 Environmental niche modelling

To determine the environmental variables influencing the present and future distribution of dwarf birch in the UK, we developed an ENM based on 763 resampled fine‐scale (≤1 km) records from the period 1960 to present. Records were sourced from national databases, conservation partners and fieldwork observations (see Borrell et al., 2018). Nineteen bioclimatic layers were obtained from the WorldClim database (http://www.worldclim.org) at 1km resolution (Hijmans, Cameron, Parra, Jones, & Jarvis, 2005), for the period 1960–1990, including 11 temperature and eight precipitation derived variables reflecting annual trends, seasonality and limiting environmental factors. High‐resolution elevation data were used to compute slope and aspect terrain characteristics using the *Raster* package (Hijmans & Etten, 2012) in R software (R Development Core Team, 2014). These variables are indicators of soil moisture, erosion, wind and solar radiation (Hoersch, Braun, & Schmidt, 2002). To avoid overfitting, we removed multiple highly correlated variables (correlation coefficient >0.7), retaining 10 for analysis (preferring less derived, e.g., annual mean temperature, rather than monthly or quarterly values; Table 1; Figure S1). Elevation was excluded due to its high correlation with temperature (Parolo, Rossi, & Ferrarini, 2008). Temperature was retained because it captures the projected change in climate change models, whilst elevation does not. All retained variables were standardized to a mean of zero and unit variance. Eight further data sets consisting of the same retained variables were generated under four representative concentration pathways (RCP) defined by the Intergovernmental Panel on Climate Change Fifth Assessment (IPCC, 2014a) at each of two future time points (2045–2065 and 2081–2100). These projections allow estimation of future temperature and precipitation values across the study area derived from the Community Climate System Model (Gent et al., 2011; Table S1).

**Table 1:** Contribution of retained environmental variables to the dwarf birch environmental niche model (ENM) and the number of environmentally associated loci detected

Variable	Description	Correlated variables^a^	ENM per cent contribution^b^	GEA loci	GEA loci (inc. cor.)^c^
AMTemp	Annual mean temperature	MTColdQ, MTColdM	34.9	17	64
MTWarmM	Max temperature of warmest month	MTWarmQ	22.1	2	6
MDR	Mean diurnal range	—	14.8	71	71
ISO	Isothermality	—	14.6	11	11
APrec	Annual precipitation	PColdQ, PWetM, PSeason, PWetQ, PWarmQ, PDryM, PDryQ	7.3	2	21
Slope	Slope	—	2.8	7	7
MTDryQ	Mean temperature of driest quarter	—	1.6	7	7
TS	Temperature seasonality	ATempR	1.4	1	3
MTWetQ	Mean temperature of wettest quarter	—	0.3	7	7
Aspect	Aspect	—	0.2	4	4

a Correlated variables include mean temperature of the coldest quarter (MTColdQ); minimum temperature of the coldest month (MTColdQ); mean temperature of warmest quarter (MTWarmQ); precipitation of coldest quarter (PColdQ); precipitation of wettest month (PWetM); precipitation seasonality (Pseason); precipitation of wettest quarter (PWetQ); precipitation of the warmest quarter (PWarmQ); precipitation of driest month (PDryM); precipitation of driest quarter (PDryQ); and annual temperature range (ATempR).

b Percentage contribution is calculated as the increase in regularized gain added to the contribution of the corresponding variable over each iteration of the model.

c Total number of SNPs associated with both the retained variable and related highly correlated variables that were excluded from the ENM analysis.

The ENMs were generated using MaxEnt (Phillips, Anderson, & Schapire, 2006) within the *dismo* package (Hijmans, Philips, Leathwick, & Elith, 2011). We performed 50 randomly subsampled replicate runs with 25% of observations retained for cross‐validation. Models were further evaluated using a binomial test of omission rate and area under the receiver operating characteristic curve (AUC). A species occurrence threshold to assess changes in occupied area was defined by “maximum training sensitivity plus specificity,” which optimizes the trade‐off between commission and omission errors (Liu, Newell, & White, 2016). Rank and percentage contribution of environmental variables is reported here, as these have been demonstrated to capture biologically important factors (Searcy & Shaffer, 2016).

### 2.2 Phenotypic data and habitat suitability projections

We identified 29 dwarf birch populations that encompass the extant UK range (Table 2; Figure S2). To test the performance of our ENM, we collected extensive phenotypic measurements of traits related to reproductive output and fitness in 20–30 individuals per population in June‐August 2013. These included the following: the number of male and female catkins, plant area, plant height and diameter of the largest stem. Cambial tissue samples were retained for genetic analysis. A subset of 18 populations was also tested for seed viability in germination experiments, a fitness proxy relevant to population persistence (Alsos, Spjelkavik, & Engelskjøn, 2003). Seed were collected in late summer, over‐wintered at 4°C and then kept in moist conditions at 18–20°C with a 14‐hr photoperiod for 60 days the following spring. For nine of these populations, 100‐day survival of seedlings during the following spring was measured (see Supplementary Materials for details).

**Table 2:** Summary information for 29 dwarf birch populations, including the number of genotyped and phenotyped individuals, habitat suitability (HSI)

Location	Pop.	Lat.	Long.	Elev. (m)	Genotyped	Phenotyped	HSI	c‐RONA	ShapleyNEUTRAL
Ben Loyal	BL	58.4	−4.4	300	6	30	0.38	0.194	0.011
Meall Odhar	MO	58.16	−4.42	404	6	29	0.45	0.168	0.006
Beinn Enaiglair	BE	57.79	−5.01	480	5	27	0.37	0.479	0.01
Luichart	LH	57.72	−4.9	268	6	29	0.54	0.131	0.008
Ben Wyvis W	BW	57.65	−4.6	482	5	30	0.77	0.149	0.01
Ben Wyvis E^a^	DG	57.65	−4.56	472	‐	21	0.75	—	—
Loch Meig	ME	57.53	−4.8	450	6	26	0.57	0.128	0.005
Glen Cannich	GC	57.34	−4.86	455	6	31	0.51	0.045	0.027
Faskanyle^a^	FS	57.33	−4.85	486	‐	17	0.66	—	—
Dundreggan Excl.	DE	57.23	−4.75	448	6	30	0.81	0.174	0.009
An Suidhe	AS	57.22	−4.81	661	2	17	0.77	0.219	0.119
Beinn Bhreac	BB	57.21	−4.82	500	6	33	0.66	0.366	0.008
Portclair	PC	57.2	−4.64	478	6	38	0.54	0.081	0.008
River Avon	AV	57.14	−3.49	549	6	28	0.59	0.306	0.01
Monadhliaths	MD	57.06	−4.31	712	6	6	0.49	0.222	0.01
Meall an tslugain	SL	57.05	−3.45	633	6	31	0.59	0.085	0.035
Loch Muick E	MU1	56.92	−3.2	492	6	31	0.17	0.223	0.006
Loch Muick W	MU2	56.92	−3.21	517	6	16	0.1	0.218	0.008
Loch Laggan	LG	56.89	−4.54	364	6	33	0.35	0.064	0.007
Loch Loch	LL	56.85	−3.65	673	6	32	0.57	0.106	0.005
Ben Gullabin	BG	56.84	−3.47	594	1	7^b^	0.58	0.194	0.422
Loch Rannoch	LR	56.76	−4.42	499	6	28	0.23	0.097	0.008
Rannoch West	RW	56.65	−4.79	306	6	32	0.61	0.218	0.007
Rannoch Moor B	RB	56.6	−4.74	304	6	10	0.51	0.169	0.008
Rannoch Moor A^a^	RA	56.6	−4.74	295	‐	27	0.51	—	—
Lennox	LX	55.97	−4.28	164	2	10	0	0.241	0.102
Emblehope^b^	EM	55.24	−2.48	448	1	1^b^	0.06	0.254	0.155
Spadeadam^b^	SA	55.05	−2.57	275	1	1^b^	0.01	0.321	0.35
Teesdale^b^	TD	54.65	−2.28	499	2	2^b^	0.06	0.291	0.133

a Populations not submitted for genetic analysis, but are considered in the comparison of HSI and reproductive output.

b Populations were exhaustively sampled.

To assess change in habitat quality across the study area, we first plotted the ENM‐derived habitat suitability index (HSI) estimates for all populations under current and future conditions. Second, ENM performance was assessed using a generalized linear model with a quasipoisson error distribution to test for a relationship between present time HSI estimates and mean population catkin counts. We also tested for a relationship between HSI (explanatory variable) and mean germination rates (response variable) using a quasibinomial error distribution. Here, we are explicitly testing the hypothesis that plants displayed greater reproductive output in locations with a higher ENM‐derived HSI.

### 2.3 RAD sequencing

The genetic samples used in this study are a subset of those described in Borrell et al. (2018). Briefly, DNA was extracted from 130 individuals (Table 2) and submitted to Floragenex (Oregon, USA) for 100 bp single‐end RAD sequencing with the enzyme *PstI*. Raw reads were filtered using Stacks v1.35 (Catchen, Hohenlohe, Bassham, Amores, & Cresko, 2013) and aligned to the dwarf birch genome, retaining only reads that align uniquely (Wang et al.., 2013) using Bowtie2 (Langmead & Salzberg, 2012) and the *ref_map.pl * pipeline. SNPs were called with a minimum depth of 5, the bounded model and a minimum log likelihood of −20, with corrections made using *rxstacks*. Finally, we filtered for loci present in ≥8 populations and a minor allele frequency >0.05.

### 2.4 Genomic signatures of local adaptation

We first used BayeScan (Foll & Gaggiotti, 2008) to compare allele frequency differences among populations and identify *F*_ST_ outlier loci, so that these could be excluded for generating a null covariance matrix for Bayenv2. Analysis was performed with 50,000 iterations thinned every 10, with 20 pilot runs, a burn‐in of 50,000 iterations and other parameters at default. Whilst *F*_ST_ outliers are candidate loci of adaptation, they can also emerge because of selection due to deleterious alleles, hybrid zones and historical demography (Bierne, Roze, & Welch, 2013). Thus, relaxed BayeScan parameters allowed us to screen outlier loci prior to GEA analysis in Bayenv2 (Günther & Coop, 2013).

Bayenv2 incorporates neutral genetic structure using a covariance matrix based on neutral markers and attempts to identify correlations between outliers and environmental gradients, potentially reducing false positives (De Mita et al., 2013). Based on recommendations in François, Martins, Caye, and Schoville (2016), to further minimize false positives, we initially excluded loci detected in BayeScan to compute a null covariance matrix of relatedness between populations, over 100,000 iterations and five independent runs. We then tested all loci (including those initially identified by BayeScan) under an alternative model where allele frequencies are determined by a combination of the covariance matrix and an environmental variable. We performed our analysis independently across all environmental variables, with the expectation that correlated predictors would return subsets of the same markers. The posterior probability that a locus is under selection across each independent environmental variable was assessed using Bayes factors (BF), with log10 posterior odds ratio values >1 defined as strong support (Jeffery, 1961). We averaged BFs over independent runs as recommended by Blair, Granka, and Feldman (2014), and following Günther and Coop (2013), we retained loci as good candidates if, in addition to a high BF, they also fell in the top 10% of Spearman correlation coefficient values, to further reduce false positives. For comparison, we also independently tested for signatures of local adaptation using redundancy analysis (RDA; Forester, Lasky, Wagner, & Urban, 2018; Rellstab et al., 2015), (see Supplementary Materials) though we consider only the candidates identified using Bayenv2 in subsequent analyses.

### 2.5 Neutral and adaptive population structure

To evaluate population structure, pairwise population *F*_ST_ was computed in Arlequin v3.5.2 (Excoffier & Lischer, 2010), and performed separately for putative neutral and adaptive loci identified through GEA analysis using a method similar to that of Candy et al. (2015).

### 2.6 Gene expression

To provide an additional line of evidence on the activity of our candidate adaptive loci, we extracted up to 10,000 bp flanking each side of the candidate locus from the *B. nana* reference genome and searched for these sequences in an RNA expression database using dwarf birch tissues derived from our genome reference plant under glasshouse conditions (Wang et al., 2013). Briefly, RNA was extracted from fresh dwarf birch leaves and flowers using a modified RNAeasy Plant Mini Kit (Qiagen), incorporating additional CTAB and phenol–chloroform steps to generate 100 bp paired‐end reads with an average insert size of 280 bp (for full methods see Zohren, 2016). These were mapped to the reference genome using Trinity software (Grabherr et al., 2013).

### 2.7 Maladaptation under present and future conditions

**Figure 1:**
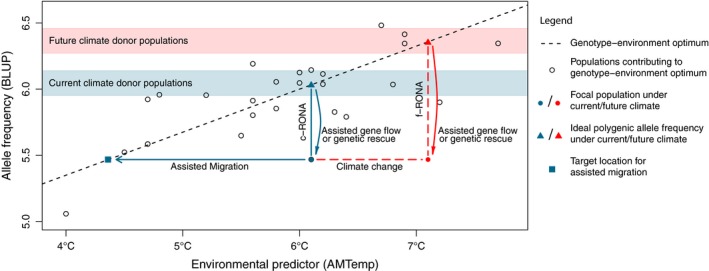
Schematic diagram of current and future risk of nonadaptedness (c‐RONA and f‐RONA), presented on a genotype–environment association (GEA) plot, where genotypes are BLUP estimates of population polygenic allele frequency for 17 loci and the environmental predictor is annual mean temperature. c/f‐RONA is the average change in allele frequency required to match our estimated optimum for current environmental conditions. Where RONA is large, we show two possible adaptation strategies; (a) assisted migration indicates the change in environmental conditions required for a population to match a genotype–environment optimum. This could take the form of a translocation of individuals to a location with a more suitable climate (e.g., a higher elevation). (b) Assisted gene flow (which in small populations is equivalent to genetic rescue) proposes movement of genetic material from a donor population with allele frequencies predicted to be better suited to the environmental conditions at the focal population. We show that the allele frequency change is likely to be larger under an example future climate scenario of 1°C warming. Blue and red bands indicate suitable candidate donor populations for assisted gene flow under current and future scenarios, respectively

We carried out RONA analysis on the nine standardized environmental variables that were associated with six or more candidate loci, allocating each locus to the single environmental variable with the largest Bayes factor (thereby avoiding double‐counting a locus in the c/f‐RONA calculations below). We estimated the vector of effect sizes, *β*, in which each value corresponds to a locus, using R package rrBLUP (Endelman 2011). In this analysis, the vector of allele frequencies *f* for each population was used as the predictor of the environment in that location. The sum of *fβ* gives an estimate of the environment (the value of the environmental variable) to which the population would be best adapted. The residual deviation of the observed value from this expectation is a measure of the deviation from the optimum environment for that population (c‐RONA) and is proportional to the change in allele frequency that would be required to match the population to its local environment (weighted by *β*). This measure is therefore analogous to those employed by Rellstab et al. (2016) and Pina‐Martins, Baptista, Pappas, and Paulo (2018), which quantify the mismatch between genotypes and environment in terms of allele frequencies. We combined information across variables by calculating the mean of the absolute residuals. Similarly, we could calculate the difference from the projected values of the environmental values under each climate change scenario to estimate f‐RONA (Figure 1).

### 2.8 Conservation prioritization

We compared the magnitude of c‐RONA across dwarf birch populations with the Shapley index (Haake et al., 2007). The Shapley index prioritizes populations based on evolutionary isolation and contribution to overall diversity based on pairwise differentiation. Several similar metrics are widely used for conservation management (Collen et al., 2011; Gumbs, Gray, Wearn, & Owen, 2018; Jetz et al., 2014). Here, we used the method outlined in Volkmann et al. (2014), which maximizes within‐species genetic diversity using a network approach implemented in NeighborNet (Bryant & Moulton, 2004; Huson & Bryant, 2006). We used linear regression to test for a relationship between absolute c‐RONA values and the Shapley index for neutral and adaptive loci.

### 2.9 Simulated assisted gene flow

For each environmental variable, and for each population in the study, we identified the population most appropriate for AGF based on the match between the local environment and the sum of *fβ*. Where several suitable populations were identified within the confidence interval of our regression, we selected the location geographically closest to the recipient population, since there could be local adaptation to undetected environmental variables (cf. Boshier et al., 2015).

### 2.10 Method validation and ENM‐GEA comparison

To validate our model, we tested the hypothesis that higher c‐RONA values would be associated with the reduced performance of fitness proxies. Therefore, we tested for a correlation between population c‐RONA values for each environmental variable, or their interactions, and (a) square root transformed catkin counts and (b) germination rate across study populations. Finally, we tested for a correlation between the relative importance of environmental variables identified in our ENM and the number of GEA loci associated with each variable.

## 3 RESULTS

### 3.1 Environmental niche models

**Figure 2:**
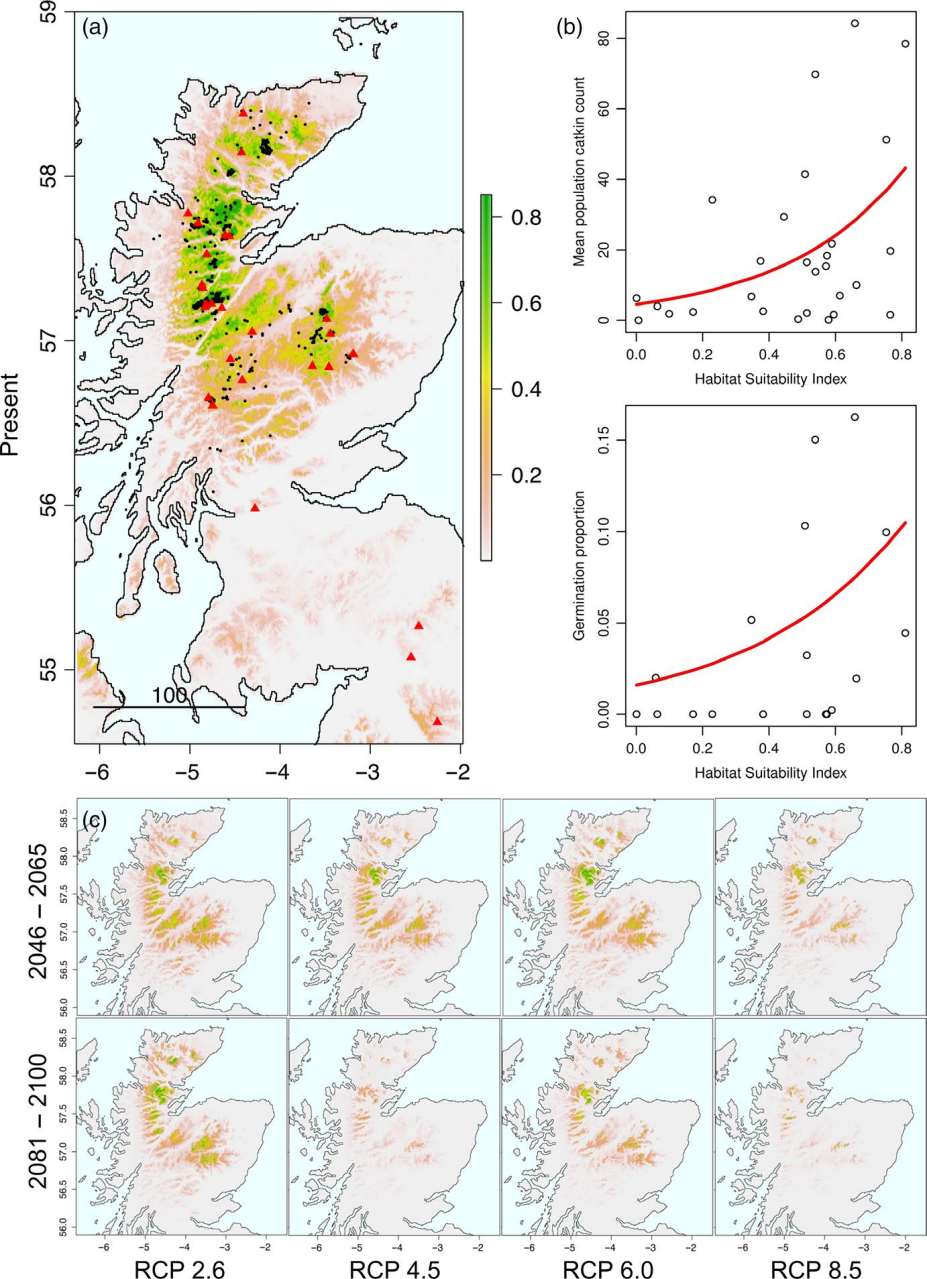
(a) Environmental niche model of dwarf birch habitat suitability (HSI) under current environmental conditions, black points indicate species distribution records and red points indicate sampled locations included in this study. (b) Regression of phenotypic fitness traits against the derived habitat suitability index. (c) Dwarf birch habitat suitability index projections under future climate scenarios

The dwarf birch ENM was well parameterized with high mean test AUC (0.946 ± 0.008) and a low mean test omission rate (0.09, *p* < .001) at a logistic threshold of occurrence of 0.193. Four variables together contributed >85% to the predictive model performance including annual mean temperature (34.9%) and maximum temperature of the warmest month (22.1%) (Table 1). The resulting model is highly concordant with qualitative field observations and inspection of variable curves showed biologically plausible responses (Figure S3). Future projections show significant declines across the species’ range with persistent populations restricted to areas of higher elevation (Figures 2 and S4). Excluding other anthropogenic pressures, under the most severe scenario (RCP8.5, 2081–2100), suitable habitat may be reduced to ~1% of the current extent (Table S2).

### 3.2 Phenotypic data and habitat suitability

Phenotypic data means are reported in Table S3. Germination success was assayed in 190 individuals and averaged 7.6% for both years with 6.1% 100‐day survival (i.e., 80% of those that germinated) with substantial variation among populations (Table S4). A single large outlier individual (Emblehope) produced an exceptionally large number of catkins strongly biasing results, thus was excluded from subsequent analysis. Present time HSI estimates for dwarf birch ranged from 0.0006 to 0.81 (Table 2), with substantial declines under all future scenarios (Figure S4). We found a significant nonlinear positive relationship between HSI and mean population catkin count (*F*_1,26_ = 7.50, *p* = .011) as well as HSI and the proportion of seeds that germinated (*F*_1,16_ = 9.52, *p* = .007; Figure 2).

### 3.3 RAD sequencing and genotype–environment associations

After quality control, RAD sequencing produced 173,460,998 reads, of which 79.1% aligned to the *B. nana* genome. Subsequently, 73.2% of aligned reads mapped to a single unique position. Three samples were excluded due to low coverage. After filtering, we retained 14,889 SNPs over 8,727 contigs. These contigs together cover approximately a third of the dwarf birch genome assembly. BayeScan identified 382 putative outlier SNPs at a conservative false discovery rate of 0.2, meant that we were more likely to remove false positives than leave false negatives. These were excluded during the generation of the Bayenv2 null covariance matrix. Subsequent GEA analysis detected 267 highly significant locus–environment associations, encompassing 303 SNPs (Table S5), with a single SNP from each locus retained for subsequent analysis. The most frequent associations were between mean diurnal range and 71 loci, and annual mean temperature and 64 loci, whereas variables such as temperature seasonality and mean temperature of driest or wettest quarters had comparatively few associated loci. Just six loci were in common between BayeScan and Bayenv2 detection methods, and BayeScan candidate loci did not report significantly higher BF scores compared to the data set as a whole. A comparison between Bayenv2 and RDA found highly significant correlation (Pearson's *r*(6) = 0.84, *p* = .008) between methods, in the number of genotype–environment associations identified for each environmental variable (Table S6; Figure S5) suggesting that both methods are identifying a similar genomic pattern of adaptation.

### 3.4 Neutral and adaptive population structure

Pairwise *F*_ST_ values between populations ranged from 0.000 to 0.701 for putative neutral markers (mean = 0.100, *n* = 14,889) and 0.000–0.260 for putative adaptive markers (mean = 0.079, *n* = 303). We found more significant pairwise *F*_ST_ values for adaptive markers (92 of 312 pairwise comparisons) than for neutral markers (49 of 312 pairwise comparisons; Table S7). We note particularly that neutral pairwise *F*_ST_ was upwardly biased by very small range edge populations (LX, EM, SA, BG and TD). If these populations are excluded, mean pairwise neutral *F*_ST_ is 0.069 and mean pairwise adaptive *F*_ST_ is 0.076.

### 3.5 Expression of putative adaptive loci

The 267 loci mapped to 185 unique scaffolds in our reference genome. Based on RNAseq data, 35 candidate regions showed evidence of gene expression in flower tissue (19%), 15 showed gene expression in leaf tissue (8%) and 13 showed gene expression in both (7%). In comparison to the overall SNP data set, we found that both flower (*X* ^2^ = 23.14, *p* < .001) and leaf (*X* ^2^ = 8.59, *p* = .003) expressed sequences are significantly over‐represented among putatively adaptive loci.

### 3.6 Potential for adaptation and conservation prioritization

**Figure 3:**
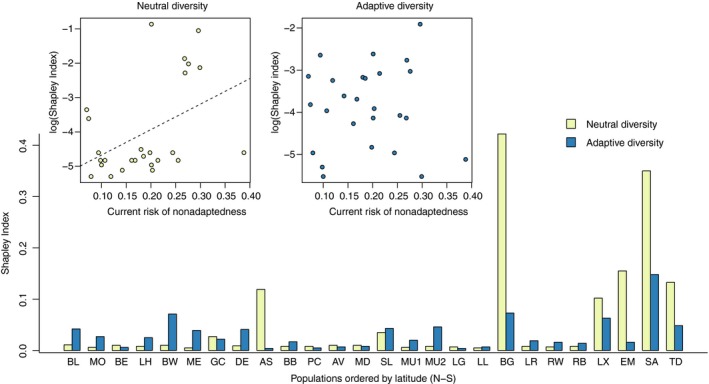
(a) Barplot of Shapley index for neutral and adaptive loci across UK *Betula nana* populations, ordered by latitude with northernmost populations to the left. Inset plots show (b) the relationship between the log‐transformed Shapley index and (c) the current risk of nonadaptedness (c‐RONA) for neutral and adaptive loci, respectively

Mean population c‐RONA based on environmentally associated SNPs under present climate was 0.22 (±0.10), ranging from 0.07 (SE ± 0.06) at Glen Cannich to 0.39 (±0.24) at Beinn Enaiglair on the Western periphery of the species range (Tables 2 and S8). BLUP estimates for all variables are presented in Figure S6. Under future climate scenarios, mean population f‐RONA was greater than c‐RONA, which increased from 0.22 (±0.10) to a maximum of 0.27 (±0.11) under scenario RCP8.5 (Table S9), with substantial variation across populations and projections. We found positive correlation between c‐RONA and the Shapley index for neutral genetic diversity (Pearson's *r*(24) = 0.44, *p* = .023), despite a number of outliers as shown by the low correlation coefficient, but no such pattern for putative adaptive genetic diversity (Pearson's *r*(24) = 0.004, *p* = .983; Figure 3). The Shapley index for neutral diversity also strongly favoured a small number of relict and range edge populations dominated by drift (e.g., BG, SA, see Borrell et al., 2018), whereas for adaptive diversity, the range of values was narrower suggested more even support across populations. Therefore, the Shapley index and our metric for maladaptation (c‐RONA) provide very different ranking for conservation value (Table 2). A consensus ranking of populations is provided in Table S10.

### 3.7 Simulating assisted gene flow

**Figure 4:**
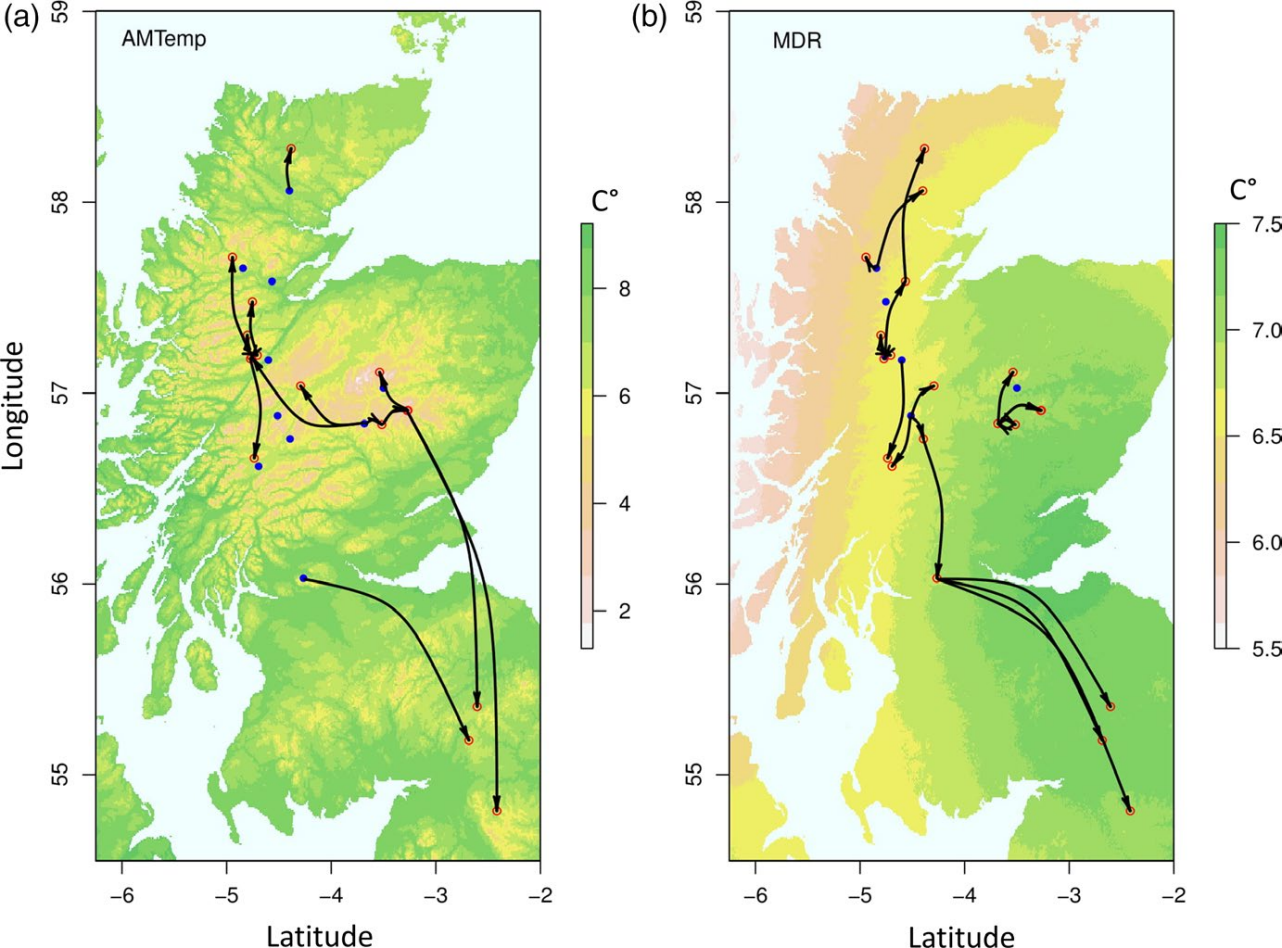
Hypothetical plots of assisted gene flow (AGF) for dwarf birch in the UK. Arrows denote movement from donor to recipient populations (red circles). Blue populations report an allele frequency close to predicted optimums, and thus, introduction of novel diversity does not decrease c‐RONA and is not required. Base maps show (a) annual mean temperature (AMTemp) and (b) mean diurnal range (MDR) environmental variables

For each population across each environmental variable, we identified the geographically closest “donor” population with an allele frequency that would reduce c‐RONA (within confidence limits) at the “recipient” site (Figures 4 and S7). This strategy proposes a pattern of dispersal from the centre of the distribution towards the periphery, particularly at the southern range edge, though there are exceptions such as transfer from the northern to southern range edge (e.g., MTColdQ, Figure S7). In some cases, the analysis does not indicate the need for AGF in particular populations, such as those at the centre of the species distribution which appear to be well matched to their environment (i.e., locally adapted).

### 3.8 Method validation and ENM‐GEA comparison

**Figure 5:**
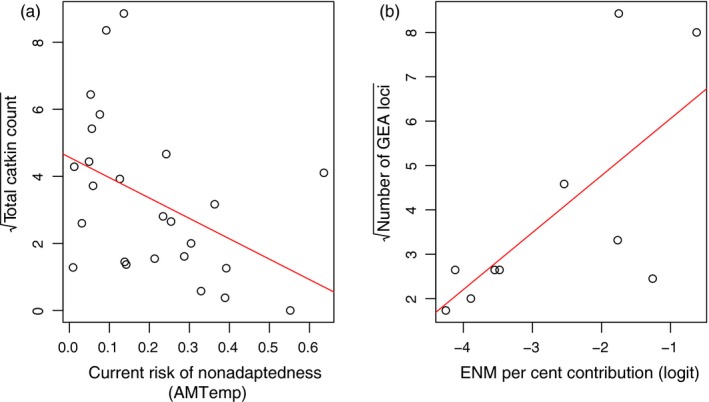
(a) The relationship between c‐RONA (for AMTemp) and mean population catkin count. (b) Correlation between the number of loci identified in genotype–environment analyses, for each environmental variable, and the corresponding percentage contribution of that variable to the environmental niche model

If c‐RONA values do indeed quantify the degree of maladaptation, they should be negatively correlated with independent measurements of population fitness. The c‐RONA values for annual mean temperature (AMTemp) were significantly negatively correlated with mean population catkin counts (*F*_1,23_ = 5.84, *p* = .025; Figure 5a) (we found a similar relationship for c‐RONA averaged across all environmental variables, data not shown). The interaction of c‐RONA for annual mean temperature and mean diurnal range correlated with germination rate (*F*_11,14_ = 8.07, *p* = .004). Finally, in a comparison of ENM and GEA methods, we found a significant correlation between the number of genotype–environment associations and the percentage contribution of environmental variables defining species range in our ENM (*r*(8) = 0.69, *p* = .027; Figure 5b).

## 4 DISCUSSION

Environmental niche modelling projects that the decline of dwarf birch across the UK is likely to continue and become increasingly severe, with almost total range loss possible by the end of the century under the highest emission scenarios. We found that catkin production and seed germination are positively correlated with ENM projections of habitat suitability. This suggests lower reproductive fitness of plants in populations with lower HSI. We cannot fully exclude the possibility that low seed germination rates are partly due to high dormancy, but it is not obvious in this context that dormancy would increase fitness. Temperature was particularly important to our ENM projections, and previous work has shown reduced production of germinable seeds by dwarf birch in warmer climates (Alsos et al., 2003). In future, an overall decline in habitat suitability across the species’ British range is likely to further reduce reproductive fitness and subsequent population persistence.

Genome‐wide analysis identified 267 significant genotype–environment associations (0.018 of loci surveyed) across 24 environmental variables, which is consistent with the number of associations identified in similar studies (Abebe et al., 2015; Manthey & Moyle, 2015; reviewed in Ahrens et al., 2018). These loci were significantly more commonly found within 10 kb of a gene annotated on our reference genome sequence with cDNA evidence for expression than were SNP loci that were not identified as candidates, increasing our confidence that candidate loci could be involved in phenotypic traits.

We observe that of the four environmental variables that contribute substantially to the dwarf birch ENM (Table 1), three of these also account for the largest number of associated loci in the genotype–environment analysis (GEA; Tables 1 and S5). Therefore, in a comparison of the two methods, we find significant agreement between ENM and GEA results in identifying important environmental variables (Figure 5b). It is not a logical necessity for environmental variables with the largest effects on species range limits to show the strongest correlation with allele frequencies. However, it is an interesting finding that suggests that we have identified biologically relevant environmental variables that influence both distribution and local adaptation of dwarf birch. It would be valuable to test for this pattern in other species, in the context of genetic models of species range limits (Polechová, 2018; Polechová & Barton, 2015).

We surveyed the allele frequencies of these GEA loci across populations to estimate c‐RONA. As expected, we find the populations which we have identified as having a poor match between genotype and environment (high c‐RONA) are particularly small or isolated, and those on the margins of the species’ distribution. This result is consistent with reconstruction of demographic history and genetic differentiation by Borrell et al. (2018), where we showed that populations with a census size of less than 10 (LX, EM, SA, BG and TD) had unusually high levels of *F*_ST_. In this previous study, we estimated the maximum likelihood value of local *F*_ST_ relative to the regional mean, using the multinomial Dirichlet likelihood function proposed by Balding and Nichols (1995) and evaluated the influence of sample size by estimating ML‐*F*_ST_ across all loci from a single individual drawn from each population (Borrell et al., 2018). From this, we concluded that these small populations were suffering from severe genetic drift. Mean pairwise *F*_ST_ for these small populations is 0.331 for neutral markers and 0.116 for putative adaptive markers, whereas for the remaining 21 populations, mean pairwise *F*_ST_ is 0.069 for neutral markers and 0.076 for adaptive markers. This suggests that in healthy populations, there is more differentiation at loci under selection, as expected. We also found that c‐RONA estimates for annual mean temperature were negatively correlated with mean population catkin counts and the interaction of c‐RONA for annual mean temperature and mean diurnal range correlated with germination rate. This suggests lower fitness due to maladaptation. Though we cannot exclude the possibility that reduced reproductive output could be an adaptive response to a poorer environment, given the short timescales involving a handful of generations, this seems unlikely.

Based on our inference that populations with low c‐RONA are more locally adapted, we then performed a comparison between c‐RONA and the Shapley index based on neutral diversity. We find that populations with the highest inferred conservation value (highest Shapley score for neutral loci) were also those with the greatest deviation from optimum allele frequencies (highest c‐RONA; Table 2; Figure 3). This implies that it may be inappropriate to use the Shapley index (and by extension, other similar metrics) based solely on neutral diversity for conservation prioritization, since this strategy would inadvertently favour poorly adapted populations that display a high degree of unique variation—in the case of dwarf birch, this is most likely due to genetic drift. Instead, we propose a conservation framework where populations with a low c‐RONA and high Shapley index based instead on adaptive diversity are prioritized. This would maximize both local adaptation and adaptive diversity, supporting future adaptive potential (Table S9).

To illustrate a possible application for this prioritization framework, we sought to identify putative dwarf birch donor populations that possess adaptive alleles at frequencies that would display reduced c‐RONA in a recipient population (Figures 4 and S7). We chose to demonstrate our approach using a current climate reference, as it could be considered more conservative, though we note that planning for future climate may have a better chance of long‐term success. In this example, our hypothetical AGF strategy involves a substantial translocation of genotypes, particularly from the centre of the range towards the periphery. Whilst controversial, AGF may be advantageous, as it can introduce or increase the frequency of preadapted alleles to allow more rapid adaptation to track changing climate, alleviate inbreeding depression or increase adaptive potential (Frankham, 2015; Prober et al., 2015); and in the process provide a demographic safeguard by augmenting population size (Hodgins & Moore, 2016). In practice, implementation of AGF is likely to take the form of composite provenancing, whereby genetic material from a combination of source populations is used (Breed, Stead, Ottewell, Gardner, & Lowe, 2013; Hodgins & Moore, 2016). This may seek to target adaptive diversity across multiple important environmental variables from across the species range, sometimes irrespective of the distance to the source population and the “local is best” paradigm (Boshier et al., 2015; Havens et al., 2015; Jones, 2013).

Our suggested approach has some limitations: RADseq only identifies variation in a subset of the genome (Lowry et al., 2016) possibly missing important adaptive loci (Harrisson et al., 2014). This concern may be addressed in future by whole‐genome population sequencing and a better understanding of the limiting returns from typing more adaptive loci (e.g., Ahrens et al., 2018). Second, our approach does not explicitly account for phenotypic plasticity or the adaptive input from new mutations (Chevin & Lande, 2011). More generally, we caution against interpreting the statistical association between the allele frequency and the bioclimatic variates (e.g., MDR) as a demonstration that the allele in question is linked to a quantitative trait locus with adaptive variation for that variable. Rather, the causal environmental variable may be unmeasured, but closely correlated with MDR. Finally, we highlight that, in our study area, the climate has been changing, albeit slowly, for several millennia, with the rate of climate change increasing more recently (Wang et al., 2014). Therefore, the clines identified here could represent adaptation to the environment of the recent past, rather than the present, and therefore may underestimate the current ecological risk. Negative density dependence could also obscure the effects of abiotic gradients. In the future, methods to accommodate change in the relative importance of environmental variables through time (Clark, Gelfand, Woodall, & Zhu, 2014) and nonlinear associations (Fitzpatrick & Keller, 2015) are likely to advance our understanding and improve estimates of local adaptation in wild populations.

## 5 CONCLUSIONS

Estimating the degree of maladaptation in populations as a criterion to inform selection of plant material for genetic rescue, composite provenancing or species reintroductions is currently the subject of considerable interest (Gibson, Espeland, Wagner, & Nelson, 2016; Leroy et al., 2018), and this is likely to increase in the context of environmental change (Aitken & Bemmels, 2016). Here we present an approach to permit rapid assessment of local adaptation and future adaptive potential in wild populations. Importantly, the estimation of maladaptation presents a testable hypothesis; specifically, that if an AGF programme translocated individuals to a site where they are expected to display reduced c‐RONA, the response of measurable fitness proxies such as catkin production should be positive. In dwarf birch, AGF would have to be combined with other management interventions focused on mitigating grazing pressure and burning to support natural regeneration, with the aim that larger populations eventually support “natural” gene flow. Similarly, AGF need not entail translocation of genetic material to an existing recipient population in the first instance. Initially, individuals of different provenance (and known allele frequencies) could be translocated to trial locations and subsequent fitness assessments would enable validation of the predicted adaptive potential. Conservationists and practitioners would then be in a better position to manage and, where appropriate, facilitate adaptation.

## CONFLICT OF INTEREST

None declared.

## Data Availability

Illumina read data from RADseq libraries have been uploaded to the European Nucleotide Archive project PRJEB26807, sample accessions ERS2598190–ERS2598376. Species records are available directly from the NBN Gateway [https://data.nbn.org.uk/]. Climate data are available from http://www.worldclim.org/
