# Supplementary material for: Genomic assessment of local adaptation in dwarf birch to inform assisted gene flow
Source: Evol Appl. 2019 Nov 24;13(1):161–75. doi: 10.1111/eva.12883 (PMC6935589; doi:10.1111/eva.12883)
Supplement: Supplementary file 1 [file EVA-13-161-s001.docx]

**Genomic assessment of local adaptation in dwarf birch to inform assisted gene flow**

**Supplementary Materials**

**Contents**

[Phenotyping and germination protocol 2](#_Toc15893978)

[Redundancy Analysis of genotype-environment associations 3](#_Toc15893979)

[Supplementary Tables 5](#_Toc15893980)

[Supplementary Figures 15](#_Toc15893981)

Phenotyping and germination protocol

All UK populations were visited once or twice in the spring and summer of 2012, 2013 or 2014, once plants were in leaf to aid identification. For each individual, the following phenotypic measurements were made:

- Latitude and longitude (GPS: Garmin Oregon 550)
- Elevation (GPS based)
- Number of male and female catkins.
- Perpendicular eight from ground level.
- Browsing pressure (percentage of browsed stems, to nearest 5%).
- Plant area (length of the longest horizontal growing axis multiplied by maximum width perpendicular to this)
- Diameter of the largest available stem at ground level.

In the years 2013 and 2014, seeds were collected from a subset of 18 populations (9 per year) in Scotland to assess germination rates. We ensured that collected catkins displayed dry brown bracts and readily dehisced to ensure maturity. Catkins were placed in labelled glassine envelopes and further air-dried for 3-5 days before being stored at 4°C for planting the following Spring. It should be noted that collected catkins would have been from the previous year’s growth, so not necessarily correlated to the female catkin count also reported in this study.

To assay germination, seeds were counted and spread on filter paper in individually labelled petri dishes. Where a large amount of seed was available for a given individual, petri dishes were replicated to avoid overcrowding. A thin layer of vermiculite was then added to prevent desiccation. Seeds were maintained at 18-20°C with a 14h photoperiod for 60 days. Germination was scored twice weekly and considered successful where a radicle ≥ 5mm was observed. For populations assayed in 2014, successfully germinated seedlings were transferred to a nutrient poor soil (similar to their preferred habitat) to assess survivability at 100 days.

Redundancy Analysis of genotype-environment associations

For comparison, we also tested the pattern of genotype-environment associations (GEA) using Redundancy Analysis (RDA), a method that has shown robust performance in scenarios of weak selection (Forester et al., 2018; Rellstab et al., 2015). RDA is a two-step analysis which extends multivariate linear regression to allow regression of multiple response variables on multiple explanatory variables. A PCA of the fitted values results in canonical axes which are linear combinations of the environmental predictors, therefore permitting identification of significant GEAs (Legendre and Legendre, 2012).

We implemented RDA in the R package vegan (Oksanen et al., 2019), using the full 14,889 SNP dataset, and a reduced set of environmental variables. Whilst we used all environmental predictor variables across independent runs of the Bayenv2 analysis (main text), here we use a reduced set of environmental variables to avoid correlated predictors being analyzed together. The reduced set of environmental variables was the same as those used for environmental niche modelling (n=10), with the additional exclusion of MTDryQ and MTWet (n=8), which showed collinearity > 0.7 in this reduced number of population sampling locations (variables for the ENM were assessed across the whole study area).

We followed the methodology outlined in (Forester et al., 2018), retaining candidate SNPs from the first three axes, with a 2.5 standard deviation significance threshold. For each candidate SNP, we first identified the environmental predictor with which it reported the highest correlation. Second, we compare candidates to those identified in the Bayenv2 GEA analysis (main text). Finally, we compare the number of SNPs associated with each environmental predictor variable across both RDA and Bayenv2 methods.

RDA identified 601 significant genotype-environment associations across eight retained predictor variables (Table S6). In a comparison of candidates between GEA methods, 11.2% of significant Bayenv2 loci were also significant in the RDA analysis. This is consistent with 9.4% of loci found in common between Bayenv2 and RDA analyses in Schweizer *et al.* (2016) and Forester *et al.* (2018). Finally, we report a highly significant correlation between the number of associations identified for each environmental variable using RDA and Bayenv2 (F_1,6_ = 14.76, p = 0.008), Figure S5). We note that this pattern was significant across a range of RDA significance thresholds, as well as with both the loci directly associated with retained variables, and the loci correlated with retained variables (see columns 5 and 6, Table 1), therefore we are satisfied it is a robust and repeatable pattern.

Supplementary Tables

**Table S1.** Projected change in global mean surface air temperature, relative to the period 1986–2005. Adapted from (IPCC, 2014b).

| Scenario | 2046-2065 | 2081-2100 |
| --- | --- | --- |
|  | Mean Δ °C (Likely range) | Mean Δ °C (Likely range) |
| RCP2.6 | +1.0 (0.4 to 1.6) | +1.0 (0.3 to 1.7) |
| RCP4.5 | +1.4 (0.9 to 2.0) | +1.8 (1.1 to 2.6) |
| RCP6.0 | +1.3 (0.8 to 1.8) | +2.2 (1.4 to 3.1) |
| RCP8.5 | +2.0 (1.4 to 2.6) | +3.7 (2.6 to 4.8) |

**Table S2.** Changes in suitable habitat area as defined by ‘maximum training sensitivity plus specificity’ threshold for dwarf birch under IPCC future climate scenarios.

| Period | Scenario | Suitable | Suitable |
| --- | --- | --- | --- |
|  |  | Area (Km^2^) | Area (%) |
| 1960-1990 | Present | 11415 | 100.00 |
| 2045-2065 | RCP2.6 | 2799 | 24.52 |
|  | RCP4.5 | 1774 | 15.54 |
|  | RCP6.0 | 2783 | 24.38 |
|  | RCP8.5 | 952 | 8.34 |
| 2081-2100 | RCP2.6 | 3021 | 26.47 |
|  | RCP4.5 | 463 | 4.06 |
|  | RCP6.0 | 1406 | 12.32 |
|  | RCP8.5 | 128 | 1.12 |

**Table S3.** Phenotypic data summary for sampled dwarf birch populations.

| Site | Pop | Lat. | Long. | Male catkins | Female catkins | Area (m^2^) | Height (cm) | Perc. browsing (%) | Stem (mm) |
| --- | --- | --- | --- | --- | --- | --- | --- | --- | --- |
| Ben Loyal | BL | 58.40 | -4.40 | 0.17 | 2.43 | 0.27 | 13.13 | 41 | 4.25 |
| Meall Odhar | MO | 58.16 | -4.42 | 1.03 | 28.34 | 0.61 | 21.69 | 16 | 3.88 |
| Beinn Enaiglair | BE | 57.79 | -5.01 | 1.52 | 15.33 | 0.23 | 24 | 31 | 5.11 |
| Luichart | LH | 57.72 | -4.90 | 1.42 | 12.39 | 0.62 | 23.52 | 40 | 5.77 |
| Ben Wyvis | BW | 57.65 | -4.60 | 3.13 | 16.57 | 0.35 | 19.77 | 19 | 4.8 |
| DJG Ben Wyvis | DG | 57.65 | -4.56 | - | - | - | - | - | 6.86 |
| Loch Meig | ME | 57.53 | -4.80 | 5.42 | 9.96 | 0.70 | 23.67 | 36 | 7.67 |
| Glen Cannich | GC | 57.34 | -4.86 | 3.84 | 37.65 | 1.33 | 26.26 | 9 | 5.69 |
| Faskanyle | FS | 57.33 | -4.85 | 32.3 | 57.19 | 0.89 | 38.1 | 12 | 5.54 |
| Dundreggan Excl. | DE | 57.23 | -4.75 | 24.8 | 56.31 | 0.92 | 28.97 | 0 | 5.5 |
| An Suidhe | AS | 57.22 | -4.81 | 0.71 | 0.88 | 0.89 | 12.76 | 6 | 4.5 |
| Beinn Bhreac | BB | 57.21 | -4.82 | 5.15 | 4.88 | 0.72 | 15.15 | 40 | 5.2 |
| Portclair | PC | 57.20 | -4.64 | 8.16 | 61.63 | 8.71 | 36.58 | 20 | 7.3 |
| River Avon | AV | 57.14 | -3.49 | 9.00 | 12.75 | 0.58 | 38.75 | 29 | 8.48 |
| Monadhliaths | MD | 57.06 | -4.31 | 0.00 | 0.33 | 1.03 | 11.0 | 25 | 4.67 |
| Meall an tslugain | SL | 57.05 | -3.45 | 1.42 | 0.23 | 0.93 | 15.08 | 51 | 3.88 |
| Loch Muick 1 | MU1 | 56.92 | -3.20 | 1.45 | 0.94 | 0.54 | 37.52 | 40 | 9.61 |
| Loch Muick 2 | MU2 | 56.92 | -3.21 | 0.69 | 1.19 | 1.25 | 50.06 | 41 | 14.59 |
| Loch Laggan | LG | 56.89 | -4.54 | 0.77 | 6.77 | 0.64 | 23.73 | 43 | 6.81 |
| Loch Loch | LL | 56.85 | -3.65 | 11.5 | 6.84 | 0.99 | 21.72 | 43 | 6.75 |
| Ben Gullabin | BG | 56.84 | -3.47 | 0.14 | 0.00 | 1.18 | 15.57 | 66 | 4.29 |
| Loch Rannoch | LR | 56.76 | -4.42 | 8.71 | 25.46 | 0.25 | 23.04 | 14 | 5.13 |
| Rannoch West | RW | 56.65 | -4.79 | 3.75 | 3.28 | 0.19 | 22.72 | 38 | 5.08 |
| Rannoch Moor B | RB | 56.60 | -4.74 | 0.00 | 2.10 | - | - | 13 | 3.89 |
| Rannoch Moor A | RA | 56.60 | -4.74 | 3.93 | 12.6 | 0.88 | 15.7 | 13 | 5.76 |
| Lennox | LX | 55.97 | -4.28 | 2.00 | 5.88 | - | 41.0 | - | 6.5 |
| Emblehope^*^ | EM | 55.24 | -2.48 | 50.0 | 300 | 25.0 | 60.0 | 10 | 15 |
| Spadeadam | SA | 55.05 | -2.57 | 0.00 | 0.00 | - | 45.0 | - | 15 |
| Teesdale | TD | 54.65 | -2.28 | 0.00 | 4.00 | - | 18.5 | 18 | 5.5 |

^*^Emblehope consisted of a single very large, presumably clonal individual, with an extremely high number of catkins. This single data point strongly influenced subsequent analyses thus it was excluded as an outlier.

**Table S4.** Germination success and survivability summary data for assayed populations.

| Year | Population | Individuals | Seeds Planted | Germinated | Germ. % | 100-Day Survivability | Surv. % |
| --- | --- | --- | --- | --- | --- | --- | --- |
| 2013 | AV | 13 | 438 | 1 | 0.23 | - | - |
| 2013 | BB | 7 | 102 | 2 | 1.96 | - | - |
| 2013 | BL | 2 | 35 | 0 | 0.00 | - | - |
| 2013 | DE | 17 | 540 | 24 | 4.44 | - | - |
| 2013 | GC | 8 | 833 | 68 | 8.16 | - | - |
| 2013 | LL | 10 | 187 | 0 | 0.00 | - | - |
| 2013 | LR | 6 | 63 | 0 | 0.00 | - | - |
| 2013 | ME | 3 | 67 | 0 | 0.00 | - | - |
| 2013 | MU | 8 | 151 | 0 | 0.00 | - | - |
| Total 2013 |  | 74 | 2416 | 95 | 3.93 | - | - |
| 2014 | DJG | 21 | 1345 | 134 | 9.96 | 27 | 2.01 |
| 2014 | FS | 23 | 492 | 89 | 18.09 | 86 | 17.48 |
| 2014 | LG | 31 | 310 | 16 | 5.16 | 15 | 4.84 |
| 2014 | LX | 5 | 230 | 0 | 0.00 | 0 | 0.00 |
| 2014 | RA | 2 | 31 | 1 | 3.23 | 0 | 0.00 |
| 2014 | RB | 3 | 21 | 0 | 0.00 | 0 | 0.00 |
| 2014 | PC | 28 | 672 | 101 | 15.03 | 77 | 11.46 |
| 2014 | TD | 2 | 14 | 0 | 0.00 | 0 | 0.00 |
| 2014 | EM | 1 | 250 | 5 | 2.00 | 1 | 0.40 |
| Total 2014 |  | 116 | 3365 | 346 | 10.28 | 206 | 6.12 |

**Table S5.** The 24 environmental variables included in this study. Uncorrelated retained environmental variables were used for Environmental Niche Modelling (ENM), whilst all variables were tested independently for genotype-environment associations (GEA).

| Variable | Description | Retained for ENM | Grouping | Bayenv2 GEA Loci  (totals inc. cor.) |
| --- | --- | --- | --- | --- |
| AMTemp | Annual Mean Temperature | X | A | 17 (64) |
| MTColdQ | Mean Temperature of Coldest Quarter |  | A | 24 |
| MTColdM | Min Temperature of Coldest Month |  | A | 23 |
| MTWarmM | Max Temperature of Warmest Month | X | B | 2 (6) |
| MTWarmQ | Mean Temperature of Warmest Quarter |  | B | 4 |
| MDR | Mean Diurnal Temperature Range | X | C | 71 (71) |
| ISO | Isothermality | X | D | 11 (11) |
| APrec | Annual Precipitation | X | E | 2 (21) |
| PWetQ | Precipitation of Wettest Quarter |  | E | 2 |
| PDryQ | Precipitation of Driest Quarter |  | E | 4 |
| PWetM | Precipitation of Wettest Month |  | E | 2 |
| PDryM | Precipitation of Driest Month |  | E | 3 |
| Pseason | Precipitation Seasonality |  | E | 1 |
| PWarmQ | Precipitation of Warmest Quarter |  | E | 4 |
| PColdQ | Precipitation of Coldest Quarter |  | E | 3 |
| Slope | Slope (derived from elevation) | X | F | 7 (7) |
| MTDryQ | Mean Temperature of Driest Quarter | X | G | 7 (7) |
| Tseason | Temperature Seasonality | X | H | 1 (3) |
| ATempR | Annual Temperature Range |  | H | 2 |
| MTWetQ | Mean Temperature of Wettest Quarter | X | I | 7 (7) |
| Aspect | Aspect (derived from elevation) | X | J | 4 (4) |
| Elev. | Elevation | - | - | 12 |
| Lat. | Latitude | - | - | 6 |
| Long. | Longitude | - | - | 48 |

**Table S6.** Comparison of GEA candidate loci identified in RDA and Bayenv2 analysis

| Variable | GEA Loci | GEA Loci (inc. cor.) | RDA (s.d. = 3) | RDA (s.d. =2.5 |
| --- | --- | --- | --- | --- |
| AMTemp | 17 | 64 | 21 | 101 |
| MTWarmM | 2 | 6 | 3 | 37 |
| MDR | 71 | 71 | 40 | 134 |
| ISO | 11 | 11 | 16 | 69 |
| APrec | 2 | 21 | 20 | 82 |
| Slope | 7 | 7 | 17 | 93 |
| MTDryQ | 7 | 7 | - | - |
| TS | 1 | 3 | 13 | 41 |
| MTWetQ | 7 | 7 | - | - |
| Aspect | 4 | 4 | 13 | 44 |

**Table S7.** Pairwise population F_ST_ for putative adaptive (upper) and putative neutral (lower) loci. Small range edge populations are highlighted in grey.

| Pop | BL | MO | BE | LH | BW | ME | GC | DE | AS | BB | PC | AV | MD | SL | MU | MU | LG | LL | BG | LR | RW | RB | LX | EM | SA | TD |
| --- | --- | --- | --- | --- | --- | --- | --- | --- | --- | --- | --- | --- | --- | --- | --- | --- | --- | --- | --- | --- | --- | --- | --- | --- | --- | --- |
| BL | - | 0.08 | 0.10 | 0.11 | 0.08 | 0.08 | 0.05 | 0.10 | 0.03 | 0.04 | 0.12 | 0.04 | 0.12 | 0.14 | 0.16 | 0.11 | 0.08 | 0.10 | 0.02 | 0.18 | 0.13 | 0.09 | 0.14 | 0.14 | 0.18 | 0.21 |
| MO | 0.06 | - | 0.04 | 0.05 | 0.07 | 0.07 | 0.08 | 0.10 | 0.00 | 0.03 | 0.07 | 0.06 | 0.02 | 0.08 | 0.10 | 0.10 | 0.01 | 0.08 | 0.05 | 0.09 | 0.09 | 0.08 | 0.10 | 0.15 | 0.16 | 0.18 |
| BE | 0.08 | 0.06 | - | 0.06 | 0.11 | 0.01 | 0.10 | 0.08 | 0.01 | 0.08 | 0.02 | 0.05 | 0.04 | 0.06 | 0.06 | 0.09 | 0.01 | 0.03 | 0.00 | 0.05 | 0.00 | 0.03 | 0.03 | 0.04 | 0.10 | 0.09 |
| LH | 0.06 | 0.05 | 0.06 | - | 0.18 | 0.09 | 0.14 | 0.10 | 0.00 | 0.13 | 0.06 | 0.05 | 0.03 | 0.08 | 0.10 | 0.07 | 0.06 | 0.05 | 0.00 | 0.05 | 0.09 | 0.05 | 0.12 | 0.13 | 0.08 | 0.15 |
| BW | 0.06 | 0.07 | 0.09 | 0.08 | - | 0.14 | 0.09 | 0.14 | 0.09 | 0.07 | 0.13 | 0.06 | 0.12 | 0.11 | 0.17 | 0.17 | 0.11 | 0.17 | 0.00 | 0.19 | 0.17 | 0.12 | 0.13 | 0.14 | 0.20 | 0.23 |
| ME | 0.07 | 0.05 | 0.06 | 0.05 | 0.07 | - | 0.08 | 0.09 | 0.03 | 0.11 | 0.11 | 0.04 | 0.10 | 0.11 | 0.13 | 0.10 | 0.06 | 0.07 | 0.00 | 0.11 | 0.07 | 0.06 | 0.14 | 0.09 | 0.20 | 0.20 |
| GC | 0.04 | 0.06 | 0.06 | 0.05 | 0.05 | 0.05 | - | 0.06 | 0.08 | 0.03 | 0.14 | 0.00 | 0.12 | 0.14 | 0.13 | 0.15 | 0.07 | 0.11 | 0.00 | 0.18 | 0.14 | 0.06 | 0.11 | 0.18 | 0.26 | 0.22 |
| DE | 0.05 | 0.07 | 0.07 | 0.06 | 0.05 | 0.06 | 0.05 | - | 0.06 | 0.07 | 0.11 | 0.02 | 0.12 | 0.14 | 0.12 | 0.19 | 0.08 | 0.09 | 0.00 | 0.15 | 0.09 | 0.10 | 0.13 | 0.18 | 0.25 | 0.18 |
| AS | 0.14 | 0.12 | 0.12 | 0.11 | 0.12 | 0.12 | 0.15 | 0.13 | - | 0.06 | 0.00 | 0.00 | 0.02 | 0.07 | 0.02 | 0.05 | 0.01 | 0.03 | 0.00 | 0.07 | 0.05 | 0.01 | 0.13 | 0.00 | 0.05 | 0.08 |
| BB | 0.04 | 0.06 | 0.08 | 0.06 | 0.05 | 0.06 | 0.03 | 0.04 | 0.11 | - | 0.10 | 0.00 | 0.09 | 0.10 | 0.12 | 0.15 | 0.06 | 0.09 | 0.00 | 0.12 | 0.10 | 0.08 | 0.09 | 0.07 | 0.22 | 0.16 |
| PC | 0.07 | 0.05 | 0.03 | 0.05 | 0.08 | 0.06 | 0.06 | 0.07 | 0.11 | 0.07 | - | 0.08 | 0.03 | 0.04 | 0.04 | 0.10 | 0.05 | 0.02 | 0.03 | 0.03 | 0.02 | 0.03 | 0.09 | 0.00 | 0.15 | 0.10 |
| AV | 0.06 | 0.07 | 0.08 | 0.07 | 0.06 | 0.08 | 0.04 | 0.04 | 0.13 | 0.03 | 0.08 | - | 0.05 | 0.07 | 0.09 | 0.09 | 0.01 | 0.07 | 0.00 | 0.12 | 0.07 | 0.05 | 0.01 | 0.16 | 0.10 | 0.10 |
| MD | 0.08 | 0.07 | 0.06 | 0.06 | 0.09 | 0.06 | 0.06 | 0.08 | 0.11 | 0.07 | 0.05 | 0.08 | - | 0.08 | 0.09 | 0.11 | 0.00 | 0.05 | 0.03 | 0.07 | 0.07 | 0.07 | 0.11 | 0.11 | 0.13 | 0.14 |
| SL | 0.09 | 0.06 | 0.06 | 0.05 | 0.08 | 0.06 | 0.09 | 0.08 | 0.17 | 0.07 | 0.05 | 0.08 | 0.05 | - | 0.05 | 0.10 | 0.02 | 0.02 | 0.15 | 0.05 | 0.09 | 0.08 | 0.10 | 0.06 | 0.26 | 0.11 |
| MU | 0.07 | 0.05 | 0.06 | 0.05 | 0.07 | 0.06 | 0.05 | 0.06 | 0.11 | 0.06 | 0.05 | 0.07 | 0.05 | 0.05 | - | 0.04 | 0.09 | 0.07 | 0.08 | 0.07 | 0.07 | 0.10 | 0.07 | 0.07 | 0.08 | 0.14 |
| MU | 0.07 | 0.05 | 0.06 | 0.05 | 0.07 | 0.06 | 0.07 | 0.07 | 0.14 | 0.06 | 0.05 | 0.07 | 0.05 | 0.07 | 0.03 | - | 0.07 | 0.07 | 0.15 | 0.08 | 0.09 | 0.04 | 0.16 | 0.04 | 0.14 | 0.16 |
| LG | 0.07 | 0.05 | 0.06 | 0.05 | 0.07 | 0.05 | 0.05 | 0.06 | 0.12 | 0.06 | 0.05 | 0.07 | 0.06 | 0.06 | 0.06 | 0.05 | - | 0.04 | 0.02 | 0.03 | 0.05 | 0.03 | 0.06 | 0.12 | 0.08 | 0.07 |
| LL | 0.07 | 0.05 | 0.05 | 0.05 | 0.08 | 0.05 | 0.05 | 0.06 | 0.10 | 0.07 | 0.05 | 0.07 | 0.05 | 0.04 | 0.05 | 0.05 | 0.05 | - | 0.00 | 0.05 | 0.00 | 0.03 | 0.09 | 0.00 | 0.15 | 0.10 |
| BG | 0.26 | 0.26 | 0.24 | 0.24 | 0.24 | 0.24 | 0.32 | 0.26 | 0.40 | 0.22 | 0.24 | 0.24 | 0.24 | 0.36 | 0.24 | 0.28 | 0.24 | 0.22 | - | 0.10 | 0.06 | 0.00 | 0.00 | 0.19 | 0.21 | 0.11 |
| LR | 0.08 | 0.06 | 0.06 | 0.05 | 0.07 | 0.06 | 0.07 | 0.07 | 0.14 | 0.06 | 0.05 | 0.07 | 0.06 | 0.07 | 0.05 | 0.05 | 0.06 | 0.05 | 0.29 | - | 0.04 | 0.05 | 0.14 | 0.00 | 0.17 | 0.08 |
| RW | 0.08 | 0.06 | 0.06 | 0.06 | 0.09 | 0.06 | 0.08 | 0.08 | 0.12 | 0.07 | 0.05 | 0.08 | 0.05 | 0.06 | 0.05 | 0.06 | 0.06 | 0.05 | 0.28 | 0.05 | - | 0.04 | 0.06 | 0.00 | 0.07 | 0.13 |
| RB | 0.06 | 0.06 | 0.05 | 0.05 | 0.07 | 0.05 | 0.07 | 0.06 | 0.13 | 0.06 | 0.06 | 0.07 | 0.05 | 0.07 | 0.05 | 0.05 | 0.05 | 0.04 | 0.29 | 0.06 | 0.05 | - | 0.12 | 0.15 | 0.10 | 0.14 |
| LX | 0.13 | 0.12 | 0.12 | 0.12 | 0.13 | 0.12 | 0.16 | 0.13 | 0.21 | 0.11 | 0.11 | 0.13 | 0.11 | 0.16 | 0.11 | 0.14 | 0.12 | 0.10 | 0.36 | 0.14 | 0.12 | 0.13 | - | 0.18 | 0.16 | 0.06 |
| EM | 0.08 | 0.05 | 0.01 | 0.00 | 0.04 | 0.03 | 0.16 | 0.09 | 0.25 | 0.03 | 0.01 | 0.05 | 0.01 | 0.15 | 0.00 | 0.06 | 0.03 | 0.00 | 0.67 | 0.07 | 0.04 | 0.09 | 0.19 | - | 0.25 | 0.04 |
| SA | 0.23 | 0.20 | 0.16 | 0.17 | 0.21 | 0.19 | 0.31 | 0.24 | 0.35 | 0.18 | 0.17 | 0.21 | 0.16 | 0.29 | 0.16 | 0.21 | 0.19 | 0.16 | 0.69 | 0.23 | 0.19 | 0.25 | 0.31 | 0.70 | - | 0.10 |
| TD | 0.17 | 0.15 | 0.15 | 0.13 | 0.17 | 0.14 | 0.20 | 0.16 | 0.23 | 0.15 | 0.13 | 0.16 | 0.13 | 0.17 | 0.13 | 0.15 | 0.15 | 0.12 | 0.38 | 0.16 | 0.15 | 0.16 | 0.21 | 0.22 | 0.32 | - |

**Table S8.** Current risk of non-adaptedness across all genotype-environment analyses for retained environmental variables.

| pop | AMTemp | MDR | ISO | MTColdM | MTWetQ | MTDryQ | MTColdQ | Slope | Elev. | Combined |
| --- | --- | --- | --- | --- | --- | --- | --- | --- | --- | --- |
| BL | 0.287 | 0.188 | 0.216 | 0.374 | 0.349 | 0.021 | 0.079 | 0.091 | 0.011 | 0.18 |
| MO | 0.056 | 0.232 | 0.237 | 0.117 | 0.167 | 0.251 | 0.156 | 0.043 | 0.018 | 0.14 |
| BE | 0.636 | 0.483 | 0.46 | 0.672 | 0.135 | 0.222 | 0.637 | 0.23 | 0.015 | 0.39 |
| LH | 0.059 | 0.069 | 0.48 | 0.307 | 0.009 | 0.39 | 0.057 | 0.131 | 0.006 | 0.17 |
| BW | 0.049 | 0.283 | 0.318 | 0.017 | 0.02 | 0.08 | 0.028 | 0.05 | 0.003 | 0.09 |
| ME | 0.126 | 0.135 | 0.081 | 0.078 | 0.013 | 0.383 | 0.22 | 0.015 | 0.018 | 0.12 |
| GC | 0.053 | 0.017 | 0.077 | 0.082 | 0.064 | 0.196 | 0.006 | 0.157 | 0.013 | 0.07 |
| DE | 0.136 | 0.159 | 0.353 | 0.048 | 0.114 | 0.373 | 0.323 | 0.144 | 0.012 | 0.18 |
| AS | 0.392 | 0.085 | 0.144 | 0.347 | 0.02 | 0.559 | 0.267 | 0.843 | 0.032 | 0.30 |
| BB | 0.363 | 0.469 | 0.3 | 0.337 | 0.056 | 0.146 | 0.516 | 0.106 | 0.005 | 0.26 |
| PC | 0.092 | 0.058 | 0.071 | 0.166 | 0.044 | 0.297 | 0.036 | 0.115 | 0.001 | 0.10 |
| AV | 0.242 | 0.317 | 0.348 | 0.464 | 0.014 | 0.268 | 0.443 | 0.08 | 0.016 | 0.24 |
| MD | 0.329 | 0.125 | 0.078 | 0.377 | 0.061 | 0.09 | 0.554 | 0.162 | 0.006 | 0.20 |
| SL | 0.009 | 0.117 | 0.111 | 0.09 | 0.033 | 0.102 | 0.067 | 0.065 | 0.032 | 0.07 |
| MU1 | 0.213 | 0.311 | 0.561 | 0.09 | 0.012 | 0.536 | 0.066 | 0.027 | 0.009 | 0.20 |
| MU2 | 0.142 | 0.314 | 0.55 | 0.036 | 0.089 | 0.161 | 0.063 | 0.566 | 0.003 | 0.21 |
| LG | 0.031 | 0.05 | 0.164 | 0.031 | 0.076 | 0.292 | 0.027 | 0.226 | 0.001 | 0.10 |
| LL | 0.012 | 0.149 | 0.23 | 0.026 | 0.005 | 0.026 | 0.161 | 0.075 | 0.025 | 0.08 |
| BG | 0.389 | 0.204 | 0.05 | 0.041 | 0.063 | 0.769 | 0.23 | 0.039 | 0.026 | 0.20 |
| LR | 0.076 | 0.125 | 0.141 | 0.051 | 0.003 | 0.53 | 0.02 | 0.013 | 0.001 | 0.11 |
| RW | 0.254 | 0.258 | 0.109 | 0.309 | 0.2 | 0.509 | 0.099 | 0.072 | 0.002 | 0.20 |
| RB | 0.138 | 0.16 | 0.402 | 0.253 | 0.063 | 0.155 | 0.159 | 0.112 | 0.007 | 0.16 |
| LX | 0.234 | 0.17 | 0.561 | 0.22 | 0.325 | 0.455 | 0.41 | 0.046 | 0.001 | 0.27 |
| EM | 0.383 | 0.292 | 0.479 | 0.128 | 0.175 | 0.756 | 0.109 | 0.084 | 0.008 | 0.27 |
| SA | 0.552 | 0.317 | 0.54 | 0.541 | 0.023 | 0.464 | 0.109 | 0.107 | 0.011 | 0.30 |
| TD | 0.304 | 0.211 | 0.149 | 0.558 | 0.174 | 0.55 | 0.491 | 0.038 | 0.008 | 0.28 |

**Table S9.** Risk of non-adaptedness under current and future climate scenarios, excluding associations with altitude and slope.

| Pop | c-RONA | f-RONA (2045-2065) | | | | f-RONA (2081-2100) | | | |
| --- | --- | --- | --- | --- | --- | --- | --- | --- | --- |
|  | (no-elev/slope) | RCP2.6 | RCP4.5 | RCP6.0 | RCP8.5 | RCP2.6 | RCP4.5 | RCP6.0 | RCP8.5 |
| BL | 0.216 | 0.272 | 0.311 | 0.262 | 0.375 | 0.280 | 0.236 | 0.254 | 0.242 |
| MO | 0.174 | 0.243 | 0.286 | 0.231 | 0.360 | 0.247 | 0.247 | 0.255 | 0.218 |
| BE | 0.463 | 0.455 | 0.478 | 0.458 | 0.521 | 0.454 | 0.405 | 0.483 | 0.426 |
| LH | 0.196 | 0.167 | 0.243 | 0.190 | 0.306 | 0.187 | 0.199 | 0.169 | 0.189 |
| BW | 0.113 | 0.135 | 0.205 | 0.104 | 0.218 | 0.127 | 0.089 | 0.134 | 0.103 |
| ME | 0.148 | 0.255 | 0.104 | 0.244 | 0.165 | 0.225 | 0.203 | 0.233 | 0.202 |
| GC | 0.071 | 0.182 | 0.076 | 0.159 | 0.086 | 0.188 | 0.116 | 0.188 | 0.113 |
| DE | 0.215 | 0.338 | 0.275 | 0.175 | 0.274 | 0.338 | 0.231 | 0.304 | 0.235 |
| AS | 0.259 | 0.278 | 0.220 | 0.274 | 0.223 | 0.294 | 0.267 | 0.300 | 0.305 |
| BB | 0.312 | 0.398 | 0.392 | 0.433 | 0.354 | 0.417 | 0.294 | 0.419 | 0.323 |
| PC | 0.109 | 0.117 | 0.202 | 0.107 | 0.163 | 0.114 | 0.157 | 0.112 | 0.161 |
| AV | 0.299 | 0.333 | 0.407 | 0.296 | 0.323 | 0.322 | 0.300 | 0.317 | 0.290 |
| MD | 0.230 | 0.422 | 0.374 | 0.453 | 0.344 | 0.415 | 0.214 | 0.436 | 0.226 |
| SL | 0.075 | 0.080 | 0.108 | 0.085 | 0.186 | 0.074 | 0.048 | 0.081 | 0.088 |
| MU1 | 0.255 | 0.244 | 0.210 | 0.243 | 0.320 | 0.236 | 0.228 | 0.258 | 0.240 |
| MU2 | 0.193 | 0.241 | 0.261 | 0.206 | 0.310 | 0.207 | 0.211 | 0.233 | 0.205 |
| LG | 0.096 | 0.160 | 0.136 | 0.180 | 0.135 | 0.170 | 0.123 | 0.167 | 0.133 |
| LL | 0.087 | 0.078 | 0.219 | 0.087 | 0.201 | 0.089 | 0.081 | 0.069 | 0.086 |
| BG | 0.250 | 0.254 | 0.185 | 0.251 | 0.124 | 0.237 | 0.240 | 0.238 | 0.232 |
| LR | 0.135 | 0.130 | 0.083 | 0.123 | 0.075 | 0.142 | 0.095 | 0.093 | 0.104 |
| RW | 0.248 | 0.240 | 0.236 | 0.206 | 0.243 | 0.170 | 0.212 | 0.236 | 0.263 |
| RB | 0.190 | 0.159 | 0.194 | 0.184 | 0.234 | 0.190 | 0.216 | 0.203 | 0.172 |
| LX | 0.339 | 0.523 | 0.538 | 0.497 | 0.387 | 0.535 | 0.327 | 0.523 | 0.350 |
| EM | 0.332 | 0.387 | 0.398 | 0.397 | 0.388 | 0.260 | 0.324 | 0.242 | 0.369 |
| SA | 0.364 | 0.329 | 0.360 | 0.322 | 0.333 | 0.357 | 0.374 | 0.344 | 0.375 |
| TD | 0.348 | 0.421 | 0.385 | 0.433 | 0.365 | 0.431 | 0.355 | 0.436 | 0.342 |
| Mean | 0.220 | 0.263 | 0.265 | 0.254 | 0.270 | 0.258 | 0.223 | 0.259 | 0.230 |

**Table S10.** Shapley values for neutral and putative adaptive loci, and population c-RONA ordered by rank. Final column represents a consensus ranking with Shapley Index for adaptive loci maximized and c-RONA minimized to optimize both adaptive diversity and current local adaptation.

| Pop | Shapley (neutral) | Pop | Shapley (adaptive) | Pop | c-RONA | Consensus rank |
| --- | --- | --- | --- | --- | --- | --- |
| BG | 0.422 | SA | 0.148 | GC | 0.045 | GC |
| SA | 0.350 | BG | 0.073 | LG | 0.064 | SL |
| EM | 0.155 | BW | 0.071 | PC | 0.081 | BW |
| TD | 0.133 | LX | 0.063 | SL | 0.085 | LR |
| AS | 0.119 | TD | 0.048 | LR | 0.097 | BL |
| LX | 0.102 | MU2 | 0.046 | LL | 0.106 | DE |
| SL | 0.035 | SL | 0.043 | ME | 0.128 | ME |
| GC | 0.027 | BL | 0.042 | LH | 0.131 | LH |
| BL | 0.011 | DE | 0.041 | BW | 0.149 | MO |
| AV | 0.010 | ME | 0.039 | MO | 0.168 | BG |
| BW | 0.010 | MO | 0.027 | RB | 0.169 | RB |
| MD | 0.010 | LH | 0.025 | DE | 0.174 | LX |
| BE | 0.010 | GC | 0.022 | BL | 0.194 | MU2 |
| DE | 0.009 | MU1 | 0.020 | BG | 0.194 | MU1 |
| LR | 0.008 | LR | 0.019 | MU2 | 0.218 | RW |
| BB | 0.008 | BB | 0.017 | RW | 0.218 | TD |
| RB | 0.008 | RW | 0.016 | AS | 0.219 | LL |
| PC | 0.008 | EM | 0.016 | MD | 0.222 | MD |
| MU2 | 0.008 | RB | 0.014 | MU1 | 0.223 | EM |
| LH | 0.008 | MD | 0.008 | LX | 0.241 | BB |
| RW | 0.007 | AV | 0.007 | EM | 0.254 | AV |
| LG | 0.007 | LL | 0.007 | TD | 0.291 | PC |
| MO | 0.006 | BE | 0.006 | AV | 0.306 | SA |
| MU1 | 0.006 | PC | 0.005 | SA | 0.321 | LG |
| ME | 0.005 | LG | 0.004 | BB | 0.366 | AS |
| LL | 0.005 | AS | 0.004 | BE | 0.479 | BE |

Supplementary Figures

**
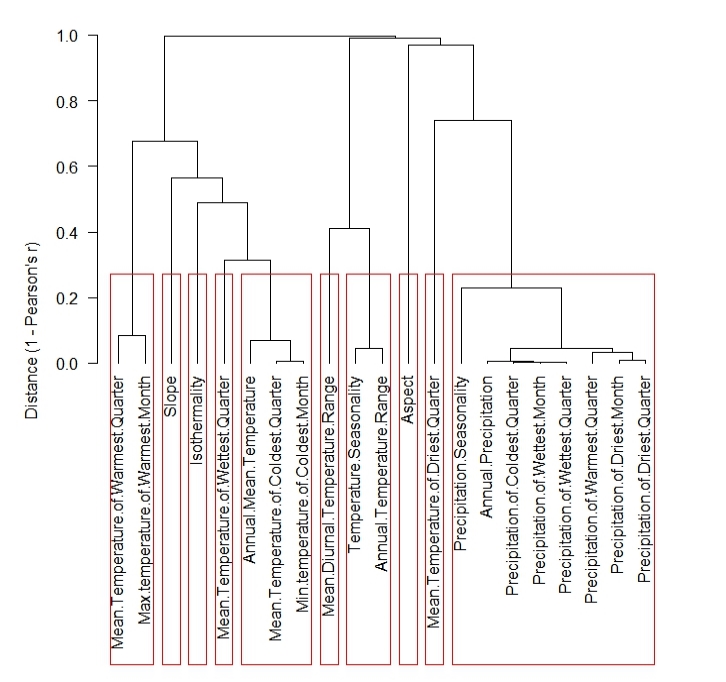
Figure S1.** Topology of collinearity between environmental variables used in this study, at a threshold of 0.7. Red boxes denote groups of retained variables (see Table S5).


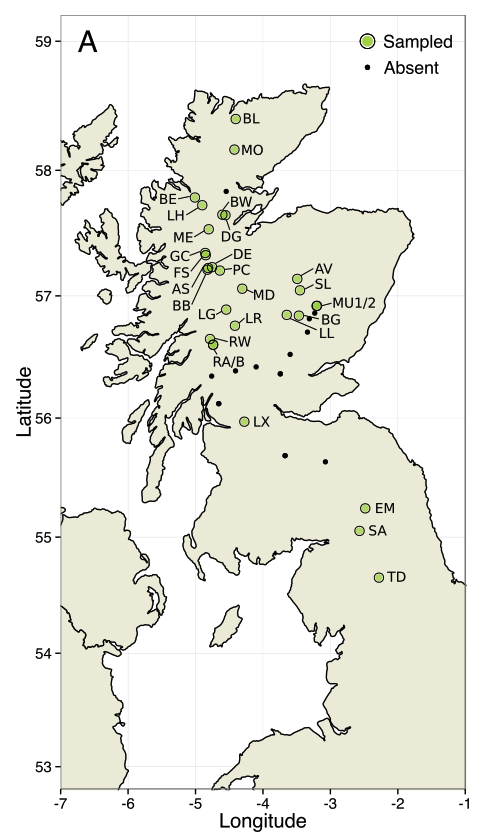


**Figure S2.** Map of dwarf birch sampling locations in the UK. Adapted from (Borrell et al., 2018).


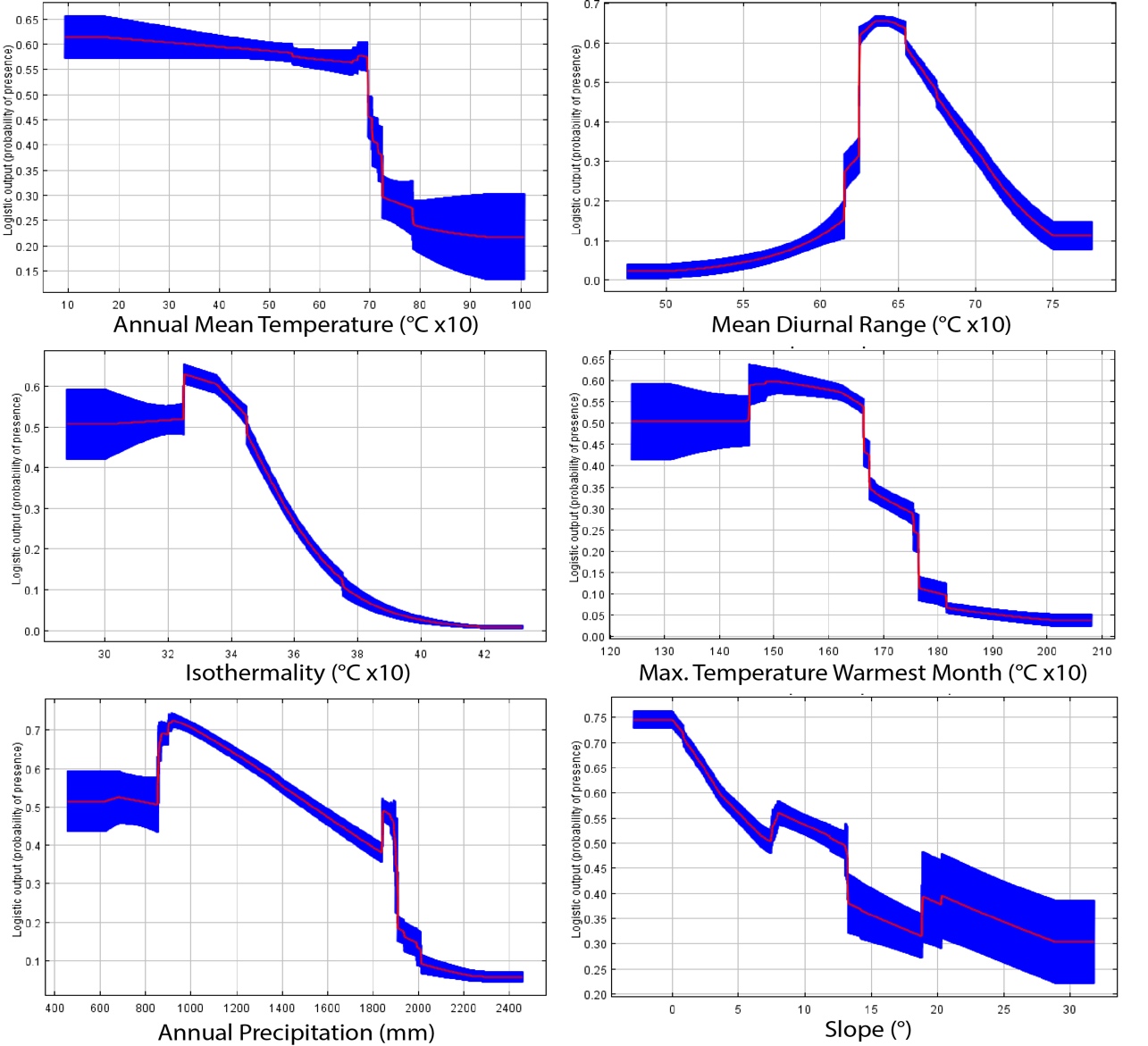

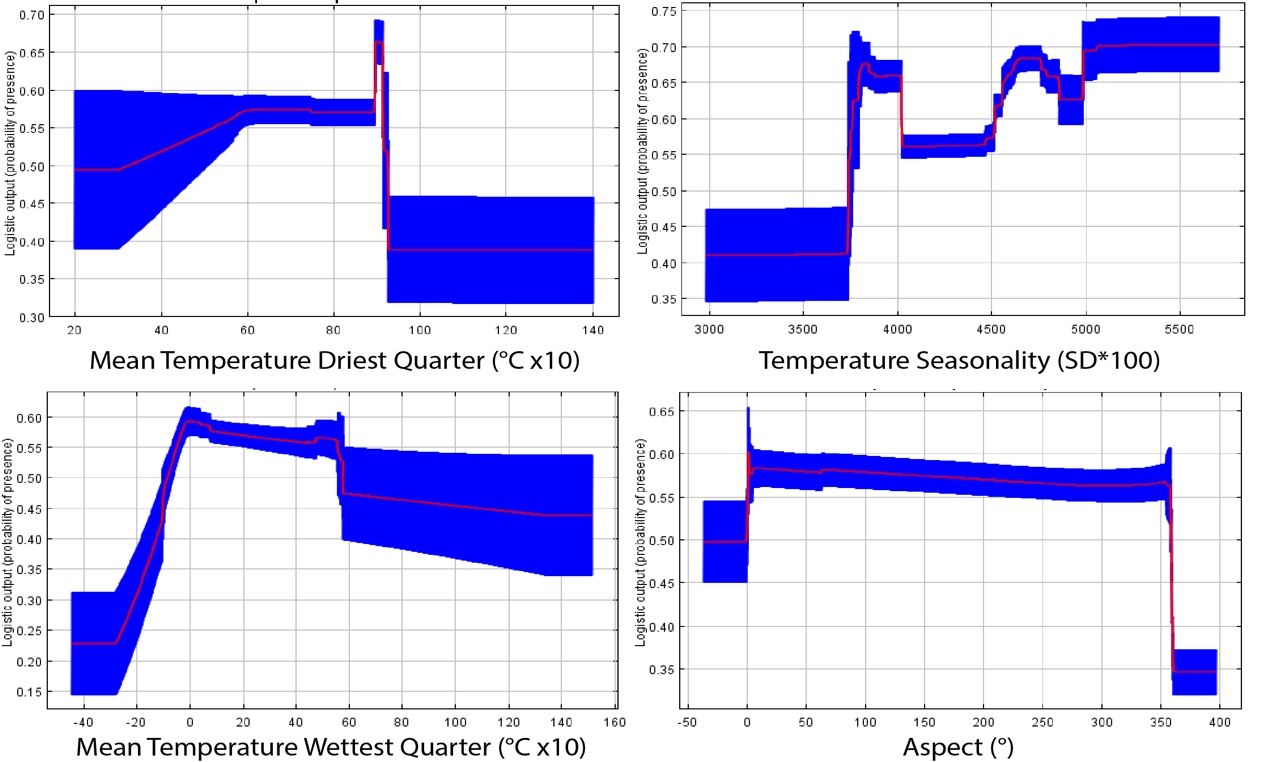


**Figure S3.** Environmental niche model variable response curves for the 10 retained environmental variables used in this study.


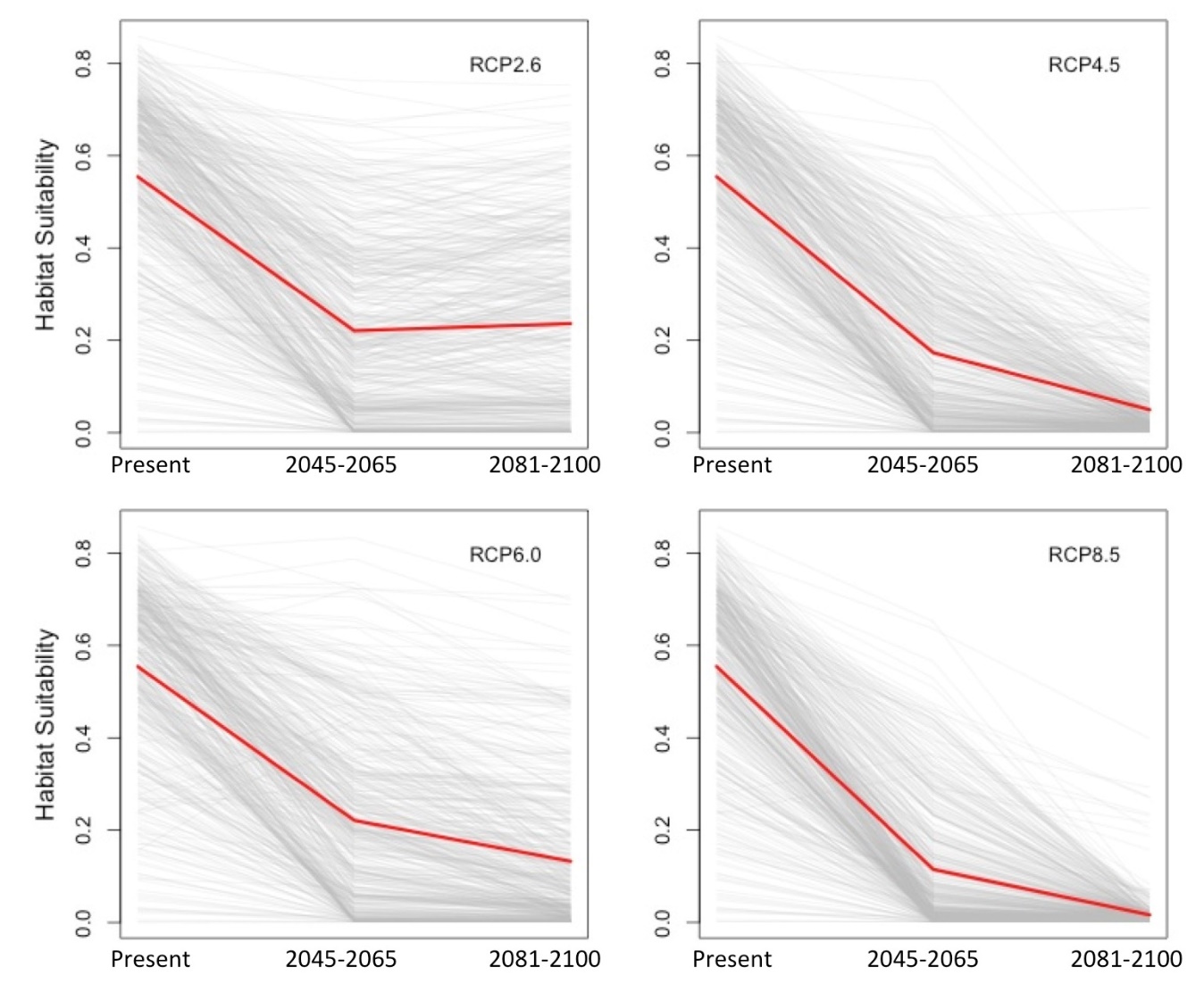


**Figure S4.** Changes in environmental niche model derived habitat suitability index (HSI) for dwarf birch under four future climate scenarios. Red line indicates overall mean for all recorded locations

**
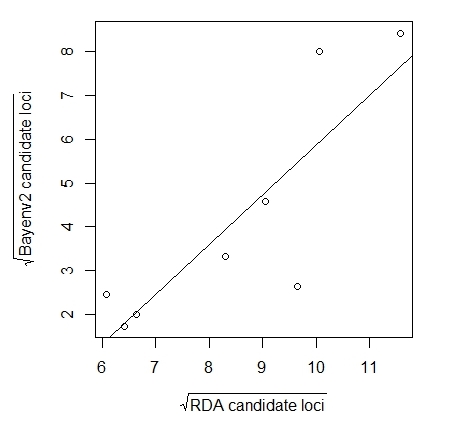
**

**Figure S5.** Comparison of the number of the number of candidate adaptive loci identified for each environmental predictor variable in RDA and Bayenv2 GEA analyses.


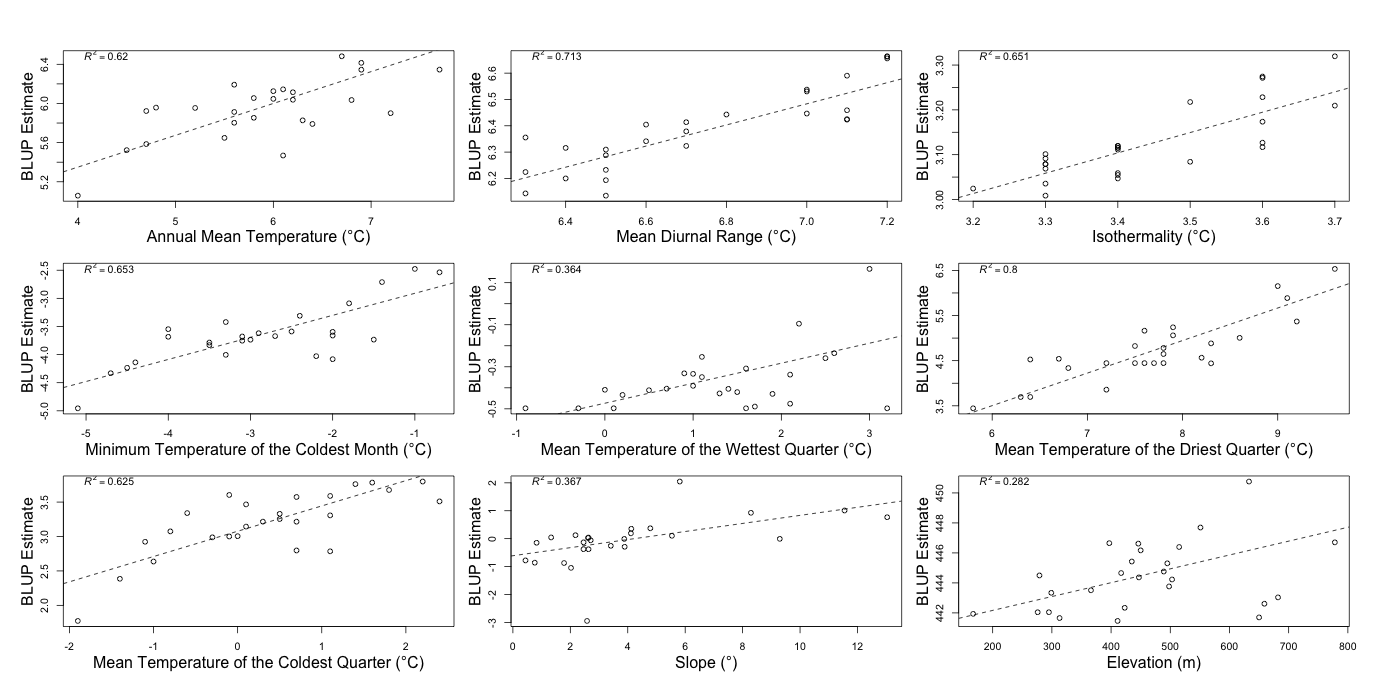


**Figure S6.** Genotype-environment association plots for nine environmental variables each with more than six associated loci, with dotted line denoting theoretical optimum genotype.


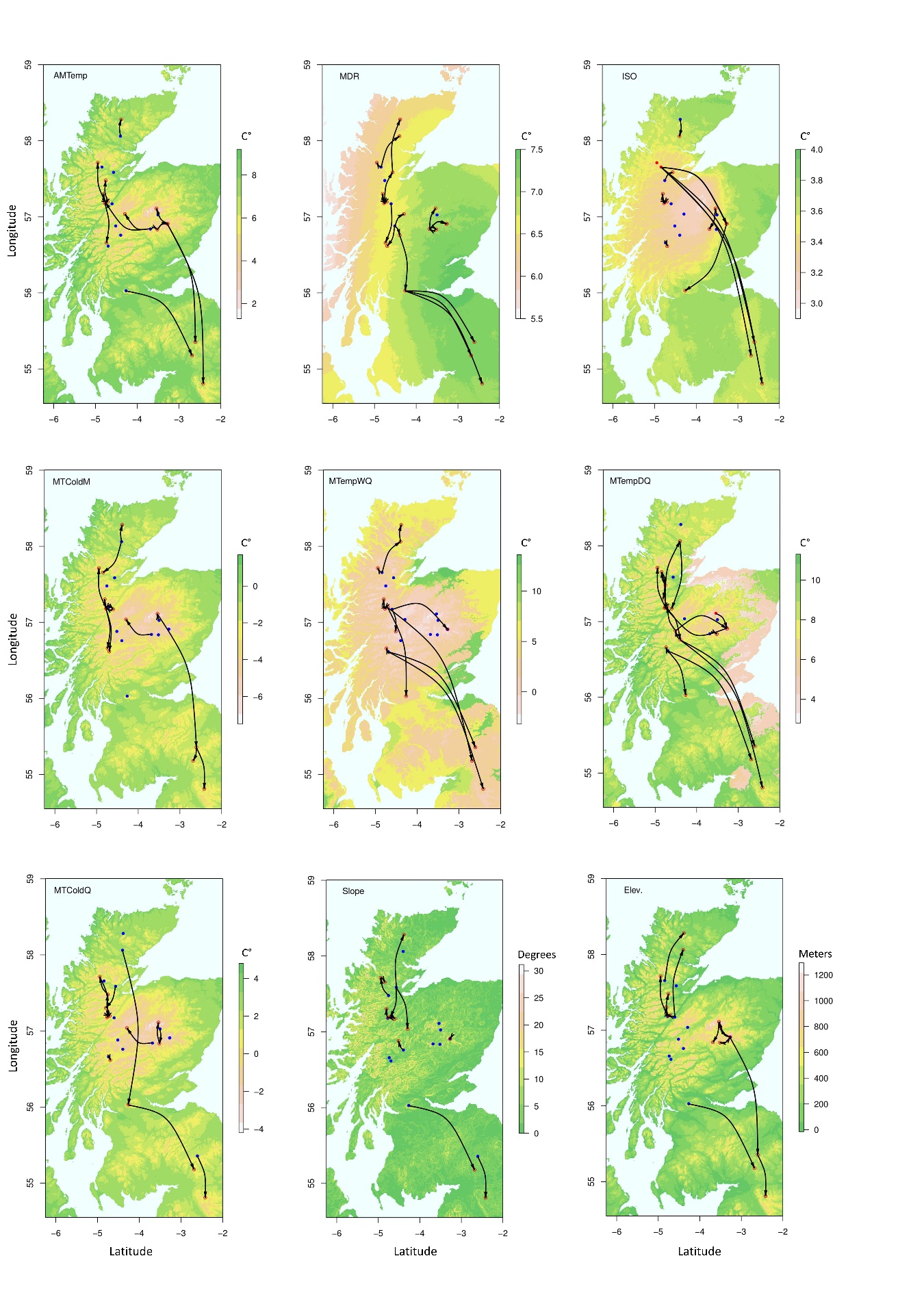


**Figure S7.** Assisted gene flow maps for nine environmental variables with more than six significantly associated loci.

**Additional References**

Borrell, J.S., Wang, N., Nichols, R.A., Buggs, R.J.A., 2018. Genetic diversity maintained among fragmented populations of a tree undergoing range contraction. Heredity (Edinb). 121, 304–318. https://doi.org/10.1038/s41437-018-0132-8

Dixon, P., 2003. VEGAN, a package of R functions for community ecology. J. Veg. Sci. 14, 927–930. https://doi.org/10.1111/j.1654-1103.2003.tb02228.x

Excoffier, L., Lischer, H.E.L., 2010. Arlequin suite ver 3.5: A new series of programs to perform population genetics analyses under Linux and Windows. Mol. Ecol. Resour. 10, 564–567. https://doi.org/10.1111/j.1755-0998.2010.02847.x

Forester, B.R., Lasky, J.R., Wagner, H.H., Urban, D.L., 2018. Comparing methods for detecting multilocus adaptation with multivariate genotype–environment associations. Mol. Ecol. 27, 2215–2233. https://doi.org/https://doi.org/10.1111/mec.14584

Legendre, P., Legendre, L., 2012. Numerical Ecology, 3rd editio. ed. Elsevier, Amsterdam.

Oksanen, J., Blanchet, F.G., Kindt, R., Legendre, P., Minchin, P.R., O’Hara, R.B., Simpson, G.L., Solymos, P., Stevens, M.H.M., Wagner, H., 2019. Package “vegan”: Community ecology package.

Rellstab, C., Gugerli, F., Eckert, A.J., Hancock, A.M., Holderegger, R., 2015. A practical guide to environmental association analysis in landscape genomics. Mol. Ecol. 24, 4348–4370. https://doi.org/10.1111/mec.13322

Schweizer, R.M., Vonholdt, B.M., Harrigan, R., Wayne, R.K., 2016. Genetic subdivision and candidate genes under selection in North American grey wolves 380–402. https://doi.org/10.1111/mec.13364
